# Anthocyanins from Rosaceae fruits: diversity, bioactivity, and potential as natural colorants

**DOI:** 10.1016/j.fochx.2026.103606

**Published:** 2026-01-29

**Authors:** Muhammad Habibul Ikhsan, Selvi Apriliana Putri, Herlandita Rona Anggraeni, Rika Septiyanti, Ari Hardianto, Jalifah Latip, Tati Herlina

**Affiliations:** a Department of Chemistry, Faculty of Mathematics and Natural Science, Universitas Padjadjaran, Sumedang, Indonesia; b Department of Chemical Sciences, Faculty of Science and Technology, Universiti Kebangsaan Malaysia, Selangor, Malaysia

**Keywords:** Anthocyanins, Rosaceae fruits, Bioactivity, Bioavailability, Extraction method, Natural food colorants, Preservation techniques

## Abstract

Anthocyanins are water-soluble flavonoid pigments responsible for red, purple, and blue hues in plant tissues. Rosaceae fruits—including blackberries, raspberries, strawberries, cherries, and apples—are rich sources of anthocyanins with notable antioxidant potential. This review highlights the diversity, extraction techniques, bioactivity, and applications of anthocyanins from Rosaceae fruits. A total of 37 compounds were identified, dominated by cyanidin, delphinidin, and pelargonidin derivatives, reflecting active biosynthetic pathways via F3′H and F3′5′H enzymes. Structural variation is influenced by glycosylation and acylation, involving glucoside, galactoside, rutinoside, and malonyl-glucoside forms. In vitro and in vivo studies demonstrate antioxidant, anti-inflammatory, antidiabetic, anti-obesity, anticancer, and antibacterial effects. Anthocyanin-rich extracts show promise as natural food colorants, offering safer alternatives to synthetic dyes. Encapsulation and copigmentation strategies further enhance their stability and functionality. These findings support the potential of Rosaceae-derived anthocyanins in health-promoting formulations and sustainable food applications

## Highlights

• Rosaceae fruits contain 37 anthocyanins with diverse chemical structures.
• Cyanidin, delphinidin, pelargonidin are dominant in Rosaceae fruits.
• Sugar and acylation patterns enhance anthocyanin structural diversity.
• Anthocyanins from Rosaceae fruits show strong bioactivity, mainly as antioxidants.
• Encapsulation and co-pigmentation improve anthocyanin colorant stability.

## Graphical abstract

**Figure:**
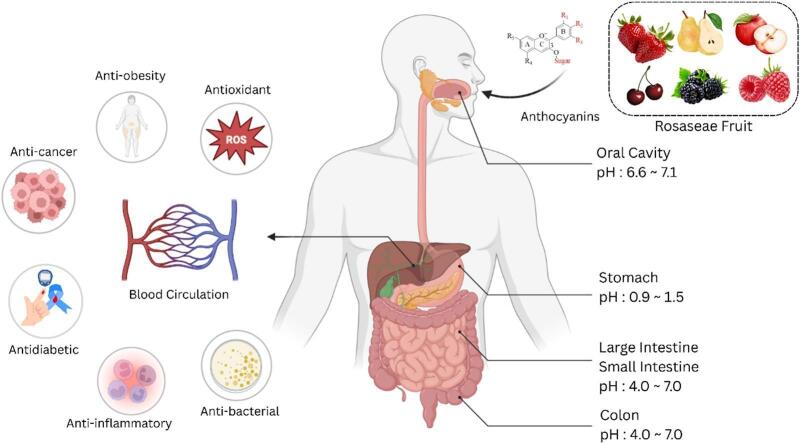

## 1 Introduction

Anthocyanins are water-soluble pigments found in the vacuoles of plants. They are part of the flavonoid group responsible for the vibrant colors such as red, purple, and blue, observed in many plant structures, including fruits, flowers, leaves, stems, tubers, and rhizomes (Meenu et al., 2023). Higher anthocyanin concentrations enhance color intensity in anthocyanin-rich fruits, vegetables, and flowers (Sharonova et al., 2021). The naming of anthocyanins is determined by the structure of the anthocyanidin core and the type of sugar moieties attached. Anthocyanidins are the aglycone forms of anthocyanins, namely cyanidin, delphinidin, pelargonidin, petunidin, peonidin, and malvidin. Their structural identity is defined by the substitution pattern of methoxy and hydroxyl groups at positions R1, R2, and R3 on the flavonoid B-ring (Rosales & Fabi, 2022). Glycosylation and acylation of anthocyanidins, typically occurring at the C3 position, result in the formation of anthocyanins. The sugar moieties commonly involved in glycosylation include glucose, rhamnose, xylose, galactose, arabinose, and rutinose (Tena & Asuero, 2022).

Structural variations in anthocyanins, primarily influenced by differences in anthocyanidin cores and sugar moieties, affect their color, chemical properties, stability, and biological activity (Tena & Asuero, 2022). These structural varitation not only affect anthocyanin coloration, solubility and resistance to environmental degradation but also modulate their biological activity. Anthocyanins exhibit a wide range of health-promoting effects, including antioxidant, anti-inflammatory, neuroprotective, anti-obesity, antidiabetic, anticancer, and cardioprotective properties (Meenu et al., 2023). Such bioactivities contribute to their therapeutic potential in preventing and mitigating chronic diseases, positioning anthocyanins as valuable compounds in functional foods and nutraceutical development.

Fruits, vegetables, and even flowers are recognized as significant sources of anthocyanins, making them important sources of these valuable bioactive components. Rosaceae family, often called the rose family, is the 19^th^ largest family of plants (Al-Mathidy et al., 2023). It comprises a wide variety of nutritious and edible fruits, such as pear (*Pyrus communis*), apple (*Malus domestica*), plum (*Prunus spinosa*), raspberry (*Rubus spp*.), sweet cherry (*Prunus avium*), peach (*Prunus persica*), and strawberry (*Fragaria x ananassa*) (Kostikova & Petrova, 2021). Studies have shown that the fruits from Rosaceae family are rich sources of phenolic compounds with significant antioxidant activity. This review will explore the diversity, extraction methods, health benefits, and applications of anthocyanins from the Rosaceae fruits, such as blackberries, raspberries, strawberries, cherries, and apples. It examines their stability, challenges in bioavailability, and future research directions to optimize their use in food, medicine, and cosmetics.

## 2 Methods

Relevant studies were retrieved from four primary databases: Google Scholar, PubMed, Scopus, and Publish or Perish. The literature search was conducted using a set of targeted keywords focusing on anthocyanin compounds derived from fruits belonging to the Rosaceae family, specifically from the genera *Rubus*, *Malus*, *Pyrus*, *Fragaria*, and *Prunus*, within the last ten years. The search results were subsequently screened to ensure alignment with the review topic. Selected studies were then systematically cited and organized for further analysis.

## 3 Structure and properties of anthocyanins

**Fig 1:**
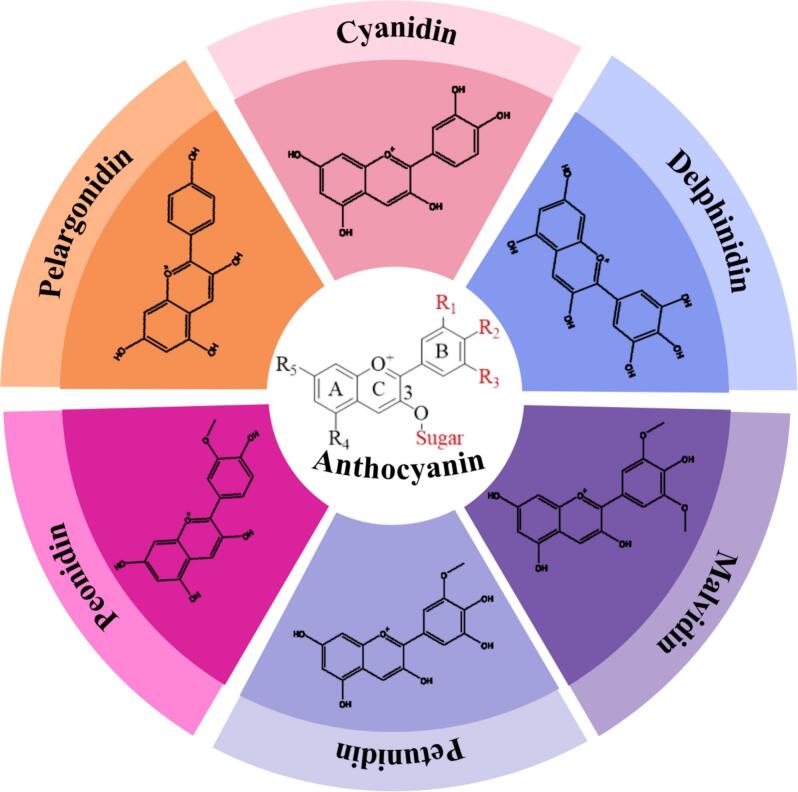
The structure of anthocyanins and anthocyanidins

Anthocyanins are polyphenolic compounds derived from anthocyanidin aglycones, which undergo glycosylation and acylation processes. Anthocyanidins, known as aglycones, are classified into 3-hydroxyanthocyanidins, 3-deoxyanthocyanidins, and O-methylated anthocyanidins. Meanwhile, anthocyanins exist as anthocyanidin glycosides or acylated anthocyanins, depending on the nature of their structural modifications (Paun et al., 2022). The classification of anthocyanins is determined by key structural factors such as the number and position of hydroxyl and methoxy groups on the anthocyanidin backbone, the type and number of sugar units linked to the aglycone, and the position of aliphatic or aromatic carboxylic acids attached to the sugar moieties (Tena & Asuero, 2022). To date, more than 500 anthocyanins have been identified. Anthocyanidins with a cyanidin-based structure are the most abundant in nature, accounting for 50%, followed by delphinidin (12%), pelargonidin (12%), peonidin (12%), malvidin (7%), and petunidin (7%) (Roy & Rhim, 2021). The structure of anthocyanins and anthocyanidins can be seen in Fig. 1.

**Fig 2:**
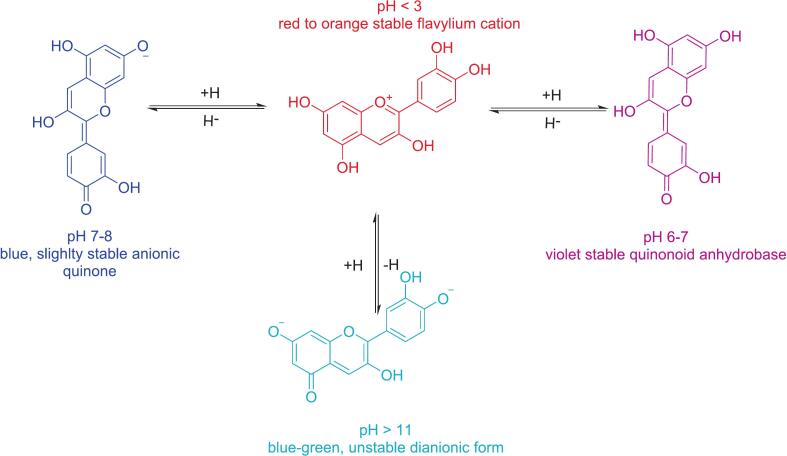
The effect of pH on anthocyanin color

The number of hydroxyl and methoxyl groups in the anthocyanin structure influences both color intensity and anthocyanin type. In addition to structural factors, pH conditions also affect anthocyanin coloration due to its ionic nature. Under acidic conditions, certain anthocyanins appear red, while at neutral pH, they exhibit a purple hue, transitioning to blue as pH increases. The red pigment in anthocyanins predominantly exists in the flavylium cation form (Prodromidis et al., 2022). The acidic properties of anthocyanins are governed by conjugated double bonds in the main ring and hydroxyl groups at C4', C5, and C7, with the C7 hydroxyl group being the most acidic. At pH ∼4, deprotonation occurs, generating a neutral quinonoid base, which is stabilized through tautomerization with the C5 hydroxyl group. As the pH increases to ∼7, the C4 hydroxyl group undergoes deprotonation, leading to the formation of an anionic base. At pH >8, further C5 deprotonation results in a dianionic base, which may transition into a chalcone anion (Tena et al., 2020). The effect of pH on anthocyanin color is illustrated in Fig. 2.

Anthocyanidins and anthocyanins are antioxidant compounds that function to prevent or inhibit oxidative stress through hydrogen donation or single-electron transfer. Their radical scavenging activity is strongly influenced by the molecular ring structure, particularly the catechol pattern on the B-ring. The catechol configuration facilitates proton and electron transfer, making compounds with this structure more effective hydrogen and electron donors due to their ability to stabilize radicals. Moreover, the catechol pattern enables the formation of ortho-semiquinone and ortho-quinone species, which further contribute to radical stabilization. In addition, the number and position of hydroxyl and methoxy groups also affect antioxidant activity. The hydroxyl group at position 3 plays a crucial role in enhancing activity through resonance mechanisms, whereas hydroxyl groups at positions 5, 7, and 4′ exert only minor effects (Ali et al., 2016).

Anthocyanins with a delphinidin backbone, characterized by three hydroxyl groups on the B-ring, exhibit superior free radical scavenging capacity compared to anthocyanins with other structural backbones. Furthermore, the type of sugar moiety bound to anthocyanins significantly influences their antioxidant potential. For instance, malvidin conjugated with arabinoside or acetyl glucoside exhibits higher IC₅₀ values, indicating lower antioxidant activity, than malvidin conjugated with glucoside or galactoside (Gui et al., 2023). In addition, the number of glycosylation sites also plays a critical role. Anthocyanins glycosylated only at the C3 position demonstrate stronger antioxidant activity than those glycosylated at both C3 and C5 positions, which is associated with a reduced ability of anthocyanin radicals to delocalize electrons (Liu et al., 2021).

## 4 Biosynthetic pathway of anthocyanin

**Fig 3:**
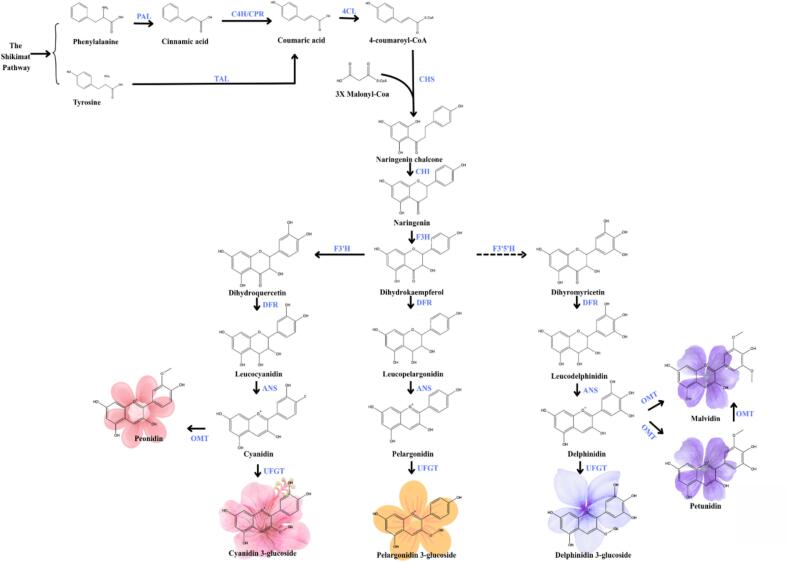
Biosynthetic pathway of anthocyanins derived from the phenylpropanoid and flavonoid pathways.

Anthocyanins are naturally occurring pigments localized within plant cells and synthesized through a complex biosynthetic pathway. These compounds belong to the flavonoid class and represent the terminal products of a specific branch within the flavonoid biosynthesis pathway, which also yields flavonols, phlobaphenes, and proanthocyanidins. Flavonols, proanthocyanidins, and anthocyanins are water-soluble and widely distributed across various plant tissues. In contrast, phlobaphenes are phenolic compounds that are soluble in alcohol but insoluble in water, and are typically produced in cereal crops such as maize, wheat, and sorghum (Cappellini et al., 2021). The biosynthetic pathway of anthocyanins is illustrated in Fig. 3.

The biosynthesis of anthocyanins occurs on the cytoplasmic surface of the endoplasmic reticulum in plant cells and begins with the conversion of phenylalanine, the primary precursor, into cinnamic acid catalyzed by the enzyme phenylalanine ammonia-lyase (PAL) (Sunil & Shetty, 2022). The resulting cinnamic acid is subsequently transformed into p-coumaric acid through the action of cinnamate-4-hydroxylase (C4H). 4-Coumaroyl-CoA is then synthesized from coumaric acid via the enzyme 4-coumaroyl:CoA ligase (4CL) (Wu et al., 2023). The next stage involves the activation of early biosynthetic genes, including chalcone synthase (CHS), chalcone-flavanone isomerase (CHI), flavanone 3-hydroxylase (F3H), flavonoid 3′-hydroxylase (F3′H), and flavonoid 3′,5′-hydroxylase (F3′5′H), which collectively contribute to the formation of dihydroflavonol compounds such as dihydrokaempferol, dihydroquercetin, and dihydromyricetin. Chalcone synthase (CHS) functions as a key catalyst in the condensation reaction between 4-coumaroyl-CoA and three molecules of malonyl-CoA, resulting in the formation of naringenin chalcone. This reaction involves the decarboxylation of malonyl-CoA, leading to the release of three molecules of CO₂ and free coenzyme A. Subsequently, naringenin chalcone is converted into naringenin by chalcone isomerase (CHI), which facilitates the intramolecular Michael addition, enabling the closure of the C-ring. This transformation alters the α,β-unsaturated double bond of the chalcone structure into a heterocyclic chroman ring, yielding a flavanone with a specific stereochemical configuration (Cappellini et al., 2021).

Naringenin, formed in the previous step through the action of chalcone isomerase (CHI), undergoes hydroxylation at the 3-position to produce dihydrokaempferol, catalyzed by flavanone 3-hydroxylase (F3H) (Sunil & Shetty, 2022). Dihydrokaempferol subsequently serves as a substrate for flavonoid 3′-hydroxylase (F3′H), leading to the formation of dihydroquercetin. This compound is further converted by flavonoid 3′,5′-hydroxylase (F3′5′H) into dihydromyricetin. In the next step, these three dihydroflavonols are reduced by dihydroflavonol-4-reductase (DFR) to yield 3,4-cis-leucoanthocyanidin, a colorless intermediate in anthocyanin biosynthesis (Sunil & Shetty, 2022).

Dihydrokaempferol, dihydroquercetin, and dihydromyricetin are subsequently converted by DFR into leucopelargonidin, leucocyanidin, and leucodelphinidin, respectively. Each of these leucoanthocyanidins serves as a direct precursor of anthocyanidins with distinct pigment colors. Leucopelargonidin is converted into pelargonidin, an anthocyanidin responsible for orange pigmentation, while leucocyanidin is converted into cyanidin, which imparts a red color. Similarly, leucodelphinidin is converted into delphinidin, which contributes to blue to purple pigmentation in plant tissues. Pelargonidin, cyanidin, and delphinidin represent the three major anthocyanidins, which are distinguished by the number of hydroxyl groups on the B-ring: pelargonidin contains one hydroxyl group, cyanidin has two, and delphinidin possesses three. In some cases, the hydroxyl groups on the B-ring undergo methylation catalyzed by methyltransferases, producing three additional anthocyanidins, namely malvidin, peonidin, and petunidin (Sunil & Shetty, 2022). The anthocyanidins produced are subsequently modified through glycosylation or acylation catalyzed by UDP-glucose:flavonoid 3-O-glucosyltransferase (UFGT) and anthocyanin acyltransferase (AAT). These modifications play a crucial role in enhancing stability, biological activity, and resistance of anthocyanins to degradation within plant tissues. The anthocyanins formed through glycosylation or acylation are then transported into the vacuole by glutathione S-transferase (GST) (Cappellini et al., 2021).

Overall, the anthocyanin biosynthetic pathway involves several key enzymes. It begins with the early phenylpropanoid pathway, where PAL, C4H, and 4CL play essential roles in generating precursor substrates. Once 4-coumaroyl-CoA is formed, the flavonoid pathway proceeds through the action of CHS, CHI, F3H, and either F3′H or F3′5′H. Notably, F3′H and F3′5′H direct the biosynthesis toward specific anthocyanidin classes, such as cyanidin and delphinidin. These intermediates are then further processed by DFR and ANS within the anthocyanidin branch. The resulting anthocyanidins undergo additional modifications by UFGT or AAT and are subsequently transported to the vacuole by GST.

## 5 Extraction methods

### 5.1 Conventional extraction techniques

Conventional extraction is one of the simplest methods, enabling the isolation of thermolabile compounds, which are sensitive to high temperatures. One of the commonly used conventional extraction methods is maceration. This technique involves grinding the sample into a coarse or powdered form, followed by soaking it in a suitable organic solvent at room temperature for at least three days, with intermittent agitation. The extraction process is conducted in a sealed container to prevent solvent evaporation (Bitwell et al., 2023). After the extraction, organic solvent is filtered from the sample, followed by concentration of the extract using vacuum evaporation. Conventional extraction methods, although easier to perform, are still primarily used on a small scale. These methods are rarely applied in industrial settings due to their low efficiency and high consumption of organic solvents. Additionally, they generate significant organic waste, posing negative environmental impacts (Mungwari et al., 2025).

### 5.2 Ultrasound assisted methods (UAE)

Ultrasound-assisted extraction (UAE) has been extensively investigated and acknowledged for its effectiveness in extraction anthocyanins from a wide range of natural matrices. This technique has been successfully applied to the extraction of anthocyanins from various sources, thereby demonstrating its versatility and consistent performance across different botanical substrates (Tena & Asuero, 2022). One of the significant advantages of UAE is its ability to improve the efficiency of anthocyanin extraction by stimulating the breaking down of plant cell walls and allowing the release of intracellular components. The implosion of these bubbles generates localized high pressure and temperature, which disrupt plant cell walls and facilitate the release of intracellular anthocyanins (Thakur et al., 2022). According to a study conducted by Hu et al. (2019), UAE yields higher recoveries of both total and individual anthocyanins compared to conventional solvent extraction techniques. This result confirms that UAE is more effective in extracting anthocyanins from natural sources than traditional methods.

In addition to its technical merits, UAE is recognized as an environmentally friendly and sustainable extraction method. As reported by Vázquez-Espinosa et al. (2019), UAE significantly reduces solvent consumption compared to conventional approaches, thereby lowering the use of organic solvents and contributing to greener processing practices. Furthermore, the extraction of anthocyanin compounds using UAE is considered more effective due to its shorter processing time compared to maceration or conventional methods. A reduced extraction duration limits the sample’s exposure to elevated temperatures, thereby minimizing the risk of thermal degradation (Tena & Asuero, 2022). Furthermore, the study conducted by Huang et al. (2025) demonstrated the effectiveness of UAE in extracting bioactive compounds from *Penthorum chinense*. The results showed that flavonoid extraction using UAE yielded the highest recovery of 241.25 ± 3.86 mg/g DW. However, to optimize the extraction process, the extraction temperature must be carefully controlled within an appropriate range, as increasing the temperature can enhance the mass transfer of flavonoids from P. chinense powder to the solvent. Nevertheless, UAE may also cause a significant rise in temperature, and prolonged extraction times could lead to the dissolution of impurities from P. chinense. In addition, excessive heat and cavitation effects may degrade unstable flavonoids.

Ultrasound-Assisted Extraction can be performed using Bath Ultrasonic Extraction (BUE) or Probe Ultrasonic Extraction (PUE), both of which are widely applied in laboratory settings (Tena & Asuero, 2022). The main difference lies in the source of ultrasonic waves: BUE employs transducers attached to the bath walls, whereas PUE utilizes a long strip transducer inserted from the top into the target solution. Probe-based ultrasonic extraction generally produces stronger ultrasonic effects than bath-based systems, as PUE delivers ultrasonic waves directly into the sample, resulting in higher extraction efficiency compared to BUE (Shen et al., 2023). In contrast, BUE is considered an indirect extraction method because ultrasonic waves must pass through two media, the water bath and the sample container before reaching the sample, thereby reducing wave intensity. Although PUE provides stronger ultrasonic effects, it is not necessarily superior to BUE, since PUE also presents limitations. The most critical drawback is the significant temperature increase during extraction, which may reduce the efficiency of bioactive compound recovery, particularly anthocyanins that are highly susceptible to thermal degradation. In practice, PUE is commonly used for small-volume samples, while BUE is more suitable for extracting larger sample quantities (Hoo et al., 2022).

According to Ordóñez-Santos et al. (2021), anthocyanin extraction from *Rubus glaucus* using UAE was 13.60% higher than the maceration technique, reducing processing time from 24 hours to 20 minutes. Similarly, as reported by Rocha et al. (2020), UAE proved to be a time-efficient method for extracting anthocyanins from red raspberry at 466 W, likely due to the ability of ultrasound waves to disrupt plant cell walls, enhancing solvent penetration and solute recovery. In the UAE process, ultrasonic power was the most influential factor affecting extraction yield, followed by solvent concentration. The authors also concluded that UAE did not cause significant anthocyanin degradation, primarily due to its shorter processing time. In addition to being applied directly, UAE can also be combined with Natural Deep Eutectic Solvents (NADES) for the extraction of anthocyanins from natural sources. Based on the study conducted by Huang et al. (2024), UAE-NADES extraction achieved a higher anthocyanin yield from *Fragaria×ananassa* Duch compared to conventional approaches. Acidified NADES composed of choline chloride–citric acid (ChCl–CA, 1:1) was selected and acidified to enhance the valorization and protection of anthocyanins through hydrogen bonding. Under the optimized conditions—ultrasonic power of 318 W, extraction temperature of 61 °C, liquid-to-solid ratio of 33 mL/g, and ultrasonic time of 19 min—the highest anthocyanin yield was obtained, reaching 1428.34 μg /g DW.

### 5.3 Microwave assisted methods (MAE)

Microwave-Assisted Extraction (MAE) is an environmentally friendly extraction method developed to address the limitations of conventional extraction techniques. MAE has proven to be highly efficient and advantageous compared to conventional extraction methods. Research has underscored multiple benefits of MAE, such as faster extraction processes, lower solvent usage, improved yields, and reduced energy consumption (Yiğit et al., 2022). MAE utilizes microwave energy to heat organic solvents, facilitating the extraction of active compounds from samples (Weremfo et al., 2020). The penetration of volume heating by microwave radiation disrupts the cellular structure. Once the cell walls are compromised, the solvent efficiently infiltrates the plant cells, facilitating the release and transport of target compounds into the extraction medium. These mechanisms contribute to a more efficient extraction, improving the release and availability of target compounds (Pham et al., 2022).

According to Kurtulbaş Şahin et al. (2021), MAE was an effective method for recovering anthocyanins from sour cherry (*Prunus cerasus* L.) peels. The optimal extraction conditions were identified as 500 W microwave power, 90 seconds of irradiation time, and 80% ethanol as the solvent. Under these parameters, the maximum yield of total anthocyanins reached 12.47 mg cyanidin-3-glucoside/g fresh matter (FM), accompanied by 69.90% DPPH inhibition, confirming strong antioxidant activity. In addition, Čechovičienė et al. (2025) reported that extraction anthocyanin from *Rubus fruticosus* L. pomace using MAE resulted in a higher total anthocyanin content (TAC) compared to UAE. These findings underscore the superior efficiency of MAE in enhancing anthocyanin recovery from blackberry pomace.

### 5.4 Pressurized liquid extraction (PLE)

Pressurized Liquid Extraction (PLE) is recognized as an environmentally friendly extraction method for obtaining bioactive compounds from plant matrices. This technique is categorized as greener compared to conventional methods because it generates small volumes of waste and can reduce both cost and time. PLE employs high pressure and temperature during the extraction process, which alters the physicochemical properties of the solvent and enables easier and deeper penetration into the sample matrix (Custodio-Mendoza et al., 2025). High pressure maintains the solvent in the liquid phase even above its boiling point, while simultaneously forcing it to penetrate the pores of the matrix. At the same time, elevated temperature decreases solvent viscosity and increases diffusivity, thereby accelerating diffusion into the solid matrix. The combination of high pressure and temperature enhances mass transfer rates, making the extraction process faster and requiring smaller amounts of solvent (Silva et al., 2024). Although high pressure and temperature are applied, the solvent conditions are carefully controlled below their critical point to ensure the liquid state is maintained. This condition is preserved to improve mass transfer by reducing solvent surface tension and viscosity, while increasing the solubility of bioactive components targeted for extraction (Andrade et al., 2021).

Due to several advantages, such as significantly faster extraction times, higher recovery rates, and lower solvent volumes, PLE has become an efficient alternative method for solid–liquid sample extraction compared to conventional techniques. This is supported by the findings of (Kitrytė et al., 2020), which demonstrated that high-pressure fractionation was more efficient in obtaining valuable constituents from blackberry pomace compared to conventional and enzyme-assisted extractions. Furthermore, PLE can also be combined with other extraction methods, such as UAE. The combination of PLE with UAE has been applied to enhance extraction yields. A study by Andrade et al. (2021) proved that PLE-UAE offers clear advantages, achieving a high total anthocyanin yield from Aronia melanocarpa pomace. Optimized conditions of 70 °C, 180 bar, a solvent concentration of 1.5% wt. citric acid, and a 200 W ultrasound bath were established. Under these conditions, 88.0% wt. of anthocyanins were extracted within 45 minutes. Additionally, a kinetic study was conducted to evaluate the influence of temperature on the total anthocyanin yield obtained under the optimal extraction conditions.

### 5.5 Deep eutectic solvents extraction

Anthocyanin extraction can be carried out using Deep Eutectic Solvents (DES) and Natural Deep Eutectic Solvents (NADES). DES is an innovative solvent system composed of two main components: a hydrogen bond donor (HBD), such as alcohols, acids, amines, amino acids, sugars, or organic acids, and a hydrogen bond acceptor (HBA), typically represented by tetraalkylammonium compounds, quaternary ammonium salts, or phosphonium salts, with choline chloride being the most commonly used HBA (Benvenutti et al., 2022). Natural components used in the preparation of DES lead to the formation of NADES, which are regarded as safer and more environmentally friendly alternatives to conventional organic solvents. NADES are typically composed of plant-derived cellular constituents, including choline derivatives, sugars, amino acids, and organic acids (Zannou & Koca, 2022).

These two solvents are considered environmentally friendly alternatives to conventional solvents due to their ease of preparation, low cost, minimal toxicity, and excellent biodegradability. Their sustainable attributes, recyclability, and compatibility with biological systems further enhance their suitability as green solvent systems. Moreover, a key advantage of DES and NADES solvents is their compatibility with advanced extraction techniques such as UAE and MAE, which can significantly enhance extraction efficiency (Meenu et al., 2023). A study by Koraqi et al. (2023) demonstrated that combination DES with UAE effectively extracted anthocyanins from raspberry fruits. The optimized conditions included a solvent volume of 35 mL, an extraction duration of 60 minutes, and the addition of 30 mL of water (v/v). These results confirmed that the UAE–DES approach is both efficient and environmentally sustainable for recovering phenolic compounds and anthocyanin constituents from raspberries. In another study reported by Kurtulbaş (2022), anthocyanin were extracted from sour cherry (*Prunus cerasus* L.) peel byproducts using a DES-based MAE system, in which the DES consisted of citric acid as the HBA and ethylene glycol as the HBD, mixed at a molar ratio of 1:4. The optimal conditions were identified as 0.1 g of peel and 50% (v/v) water addition to the DES, resulting in maximum recovery of total phenolic content (TPC) and total anthocyanins.

Although modern extraction methods such as UAE, MAE, PLE, and the use of solvents like DES and NADES provide clear advantages in terms of reduced solvent consumption, shorter extraction times, and improved environmental sustainability, studies that compare the efficiency of anthocyanin extraction from natural sources, particularly Rosaceae fruits, at an industrial scale remain scarce. Existing publications predominantly emphasize the benefits of modern extraction techniques at the laboratory scale relative to conventional methods such as maceration, yet offer limited evidence regarding their feasibility and applicability at the industrial level. This limitation highlights a research gap that warrants further investigation in future studies.

## 6 Identification of Anthocyanins from Extracts

Various techniques have been employed for the identification and characterization of anthocyanins in different matrices, including mass spectrometry (MS), nuclear magnetic resonance (NMR), and high-performance liquid chromatography (HPLC), with chromatography and spectrophotometry being the most widely applied methods (de Souza Mesquita et al., 2023). HPLC coupled with MS is the most commonly used approach due to its ability to systematically isolate, identify, and quantify individual anthocyanins within complex food matrices. This technique involves dissolving the food sample and passing it through a column containing a stationary phase, where anthocyanins are separated based on their unique interactions with the mobile phase (Custodio-Mendoza et al., 2024). In addition to HPLC, ultra-high-performance liquid chromatography (UHPLC) has been extensively applied in the past five years for the determination of anthocyanin content, as it operates at higher pressures (10,000–15,000 psi) and utilizes smaller stationary phase particles, thereby achieving greater efficiency, faster separations, improved resolution, and reduced solvent consumption (Wang et al., 2022).

In addition, both HPLC and UHPLC are frequently employed with various types of detectors, such as photodiode array (PDA), diode array detector (DAD), fluorescence, or mass spectrometry (MS). The choice of detection techniques—ranging from UV detectors to high-resolution mass spectrometry (HRMS)—is determined by the specific objectives of the analysis. UV detectors, including PDA and DAD, provide valuable insights into anthocyanin types during the profiling stage, whereas MS, particularly HRMS, is preferred for its unmatched precision in distinguishing structurally similar anthocyanin molecules, thereby offering comprehensive profiling capabilities. The combination of HPLC/UHPLC with these detectors further enhances sensitivity, specificity, and provides detailed structural information on anthocyanins present in the sample (Custodio-Mendoza et al., 2024).

For example, Bouillon et al. (2024) employed UPLC–QTOF–MS to detect anthocyanin content from *Malus domestica* Borkh. UPLC was used to separate the compounds within the sample, which were subsequently introduced into the Q-TOF mass spectrometer. These compounds underwent ionization, generating m/z values that were then compared with standards and reference data to determine the identity of the compounds present in the sample. In addition, Q-TOF was utilized to accurately measure the molecular mass of the compounds. Metabolite identification was achieved by integrating two key pieces of information: the retention time from UPLC and the m/z values obtained from Q-TOF. This study successfully confirmed the presence of cyanidin-3-O-galactoside at concentrations ranging from 0.237 to 30.947 mg/100 g FW of sample weight. In a related study, Krgović et al. (2025) detected anthocyanin compounds present in *Rubus occidentalis* L. extracts using an HPLC–DAD system with a reversed-phase RP-18 column. The first step involved the separation of extract components based on polarity differences through gradient elution with acidified water and acetonitrile as the mobile phases. During the separation process, anthocyanins were detected using a diode array detector (DAD) at 520 nm, which is the characteristic wavelength for anthocyanins. Identification was performed by comparing the retention time and UV–Vis spectra of sample peaks with the cyanidin-3-O-rutinoside reference standard. This study successfully confirmed the presence of cyanidin-3-O-rutinoside at a concentration of 760 mg/100 g DW in the sample.

## 7 Overview of anthocyanins from Rosacea fruits

The Rosaceae family, commonly known as the rose family, ranks as the 19^th^ largest plant family, encompassing approximately 100 genera and recognized as one of the most economically significant plant families, including fruit-bearing, nut-producing, ornamental, aromatic, herbaceous, and woody species (Al-Mathidy et al., 2023). This family is widely distributed across various ecosystems, making it cosmopolitan and present worldwide (Razola-Díaz et al., 2024). Fruits from the Rosaceae family typically exhibit vivid red, purple, and yellow hues, attributed to their natural pigmentation and commonly used in traditional medicine (Manzoor et al., 2023). Research indicates that these fruits are rich in phenolic compounds, exhibiting strong antioxidant properties that contribute to health benefits (Razola-Díaz et al., 2024). Their bioactive compounds provide antioxidant properties that contribute to disease prevention and overall well-being such as cancer, cardiovascular and other chronic conditions (Manzoor et al., 2023).

Fruits commonly consumed from the Rosaceae family originate from the genera *Rubus*, *Malus*, *Pyrus*, *Fragaria*, and *Prunus.* Species belonging to *Rubus* genus are commonly consumed and have long been utilized in traditional medicine worldwide. Species within the genus are rich sources of bioactive compounds from the polyphenol group, particularly phenolic acids, anthocyanins, and flavonoids (Buczyński et al., 2024). Apples, derived from the Malus genus, are among the most frequently consumed fruits worldwide. They are abundant in diverse bioactive phytochemicals and offer a wide range of health benefits, including antioxidant, anti-inflammatory, cardioprotective, and neuroprotective effects, as well as gastrointestinal support and modulation of glucose and lipid metabolism (Oyenihi et al., 2022. Another widely consumed fruit from the Rosaceae family is the pear, which belongs to the genus *Pyrus*. Pears are known to contain a diverse array of bioactive compounds, including flavonoids, tannins, alkaloids, and phenolic acids, all of which have been associated with various pharmacological activities such as anticancer, anti-inflammatory, antimicrobial, antioxidant, and estrogenic effects (Soria-Lopez et al., 2025).

Strawberries, another economically significant fruit from the Rosaceae family, belong to the genus *Fragaria* and are considered among the most important food crops globally. *Fragaria* species are extensively used in both conventional and traditional medicine, serving as rich sources of vitamins and therapeutic raw materials (Atazhanova et al., 2025). Lastly, fruits from the genus *Prunus* are also recognized for their high content of polyphenolic compounds with notable health benefits. These include phenolic acids, flavonols, and anthocyanins, which contribute to their antioxidant and therapeutic properties (Popović et al., 2021). The anthocyanin content of fruits from the Rosaceae family is summarized in Table 1.

**Table 1:** Anthocyanin content in fruits of the Rosaceae family

Plants	Extraction Method	Identification method	Antocyanins	Content	Reference
**Rubus**					
*Rubus fruticosus*	PLE • 5 grams fresh blackberry residue • Water pH 2.5 (adjusted by the direct addition of citric acid) • 80°C • Pressure 7.5 MPa • 30 min extraction	UHPLC-QTOF-MS	Cyanidin-3-*O*-glucoside	105.65 mg/100 g FW	(Machado et al., 2015)
			Cyanidin-3-*O*-rutinoside	9.54 mg/100 g FW	
			Cyanidin-3-*O*-(6”-malonylglucoside)	2.19 mg/100 g FW	
			cyanidin-3-(6”-dioxalyl-glucoside)	3.71 mg/100 g FW	
*Rubus fruticosus*	UAE • 300 mg Dried berry powder • vortexed for 3 min in 10 mL of a 9:1 mixture of aqueous formic acid (8.5 %) and acetonitrile/methanol mixture (85/15). • ultrasonication for 5 min	HPLC-PD	Cyanidin-3-*O*-glucoside	732.9 mg/100 g DW	(Lee et al., 2016)
			Cyanidin-3-*O*-rutinoside	48.4 mg/100 g DW	
*Rubus idaeus*			Cyanidin-3-*O*-sophoroside	476.4 mg/100 g DW	
			Cyanidin-3-*O*-glucoside	59.5 mg/100 g DW	
			Cyanidin-3-*O*-rutinoside	30.0 mg/100 g DW	
*Rubus fruticosus*	PLE • Ethanol 70% • pressure of 10.0 MPa • temperature of 80 °C • 30 min	UHPLC	Cyanidin-3-*O*-glucoside	163 mg/100 g DW	(Machado et al., 2017)
			Cyanidin-3-*O*-rutinoside	15 mg/100 g DW	
			Cyanidin-3-*O*-malonyl-glucoside	2 mg/100 g DW	
	UAE • Ethanol 70% • frequency and power fixed at 37 kHz and 580 W • 2 g of sample in 45 mL of solvent • sonicated for 90 min at ambient pressure and 80°C		Cyanidin-3-*O*-glucoside	205 mg/100 g DW	
			Cyanidin-3-*O*-rutinoside	17 mg/100 g DW	
			Cyanidin-3-*O*-malonyl-glucoside	3 mg/100 g DW	
	UAE + PLE • Ethanol 70% • 5 grams of sample in 4 mL of solvent • Sonicated 8 min at 80°C • PLE was performed for 30 min		Cyanidin-3-*O*-glucoside	124 mg/100 g DW	
			Cyanidin-3-*O*-rutinoside	11 mg/100 g DW	
			Cyanidin-3-*O*-malonyl-glucoside	2 mg/100 g DW	
*Rubus idaeus*	Maceration • 500 g raspberry pulp was extracted thrice with methanol/ water/formic acid (90:9:1) v/v at 4 °C	HPLC-IT-TOF	Malvidin-3-*O*-glucoside	2.70%	(Wu, Gao, et al., 2018; Wu, Yang, et al., 2018)
			Cyanidin-3-*O*-glucoside	30.01%	
			Pelargonidin-3-*O-*glucoside	41.02%	
			Peonidin-3-*O*-glucoside	26.28%	
*Rubus ulmifolius* Schott	Maceration • 1 g sample • 1 H • 30 mL of ethanol/water (80:20, v/v) containing 0.5% HCl. • After filtration (Whatman No. 4 paper), the residue was re-extracted with 30mL of ethanol/water (80:20, v/v) acidified with 0.5% HCl.	UPLC-DAD-ESI/MSⁿ system	Cyanidin-3-*O*-glucoside	1469 mg/100 g extract	(Da Silva et al., 2019)
			Pelargonidin-3-*O*-glucoside	223400 mg/100 g extract	
			Cyanidin-3-*O*-xyloside	262 mg/100 g extract	
*Rubus idaeus*	PLE • 100 g of blackberry pomace • Ethanol • 10.3 MPa • 90 °C • 45 min	UPLC-UHR-TOF	Cyanidin-3-glucoside	550.96 mg/100 g DW	(Kitrytė et al., 2020)
			Cyanidin-3-rutinoside	7.93 mg/100 g DW	
			Cyanidin-3-arabinoside	9.63 mg/100 g DW	
			Cyanidin-3-*O*-malonyl-glucoside	19.87 mg/100 g DW	
			Cyanidin 3-(6”-dioxalylglucoside)	9.51 mg/100 g DW	
			Peonidin-3-arabinoside	4.96 mg/100 g DW	
*Rubus idaeus*	UAE • 600 mg sample • 20 mL Ethanol 38% • sonication at 466 W for 16 min	HPLC-PDA	Cyanidin-3-*O*-glucoside	331 mg/100 g extract	Rocha et al., 2020)
			Cyanidin-3-*O*-sophoroside	504 mg/100 g extract	
*Rubus discolor*	UAE 10 g sample extracted with 100 ml ethanol for 1 hour in an ultrasonic bath and incubation in the dark for 24 hours after sonication	HPLC-DAD	Cyanidin-3-*O*-glucoside	3220 mg/100 g DW	(Veličković et al., 2021)
*Rubus fruticosus*	• Mature fruits of *Rubus fruticosus* L. blended to obtain a juice rich in anthocyanins • juice underwent centrifugation, filtration (Whatman No. 4), freezing, and freeze-drying (lyophilization). • The lyophilized extract was reconstituted in water to achieve a final concentration of 100 mg/mL.	UPLC-DAD-ESI/MS	Cyanidin-*O*-hexoside	376.1 mg/100 g Extract	(Vega et al., 2021)
			Cyanidin-3-*O*-glucoside	181 mg/100 g Extract	
			Cyanidin-*O*-pentoside	126.5 mg/ 100 g Extract	
*Rubus fruticosus*	UAE • 30 g frozen berry powder • 150 mL 80% methanol containing HCl 0.01% • ultrasonication for 40 min room temperature	HPLC–DAD–QTOF-MS	Cyanidin-3-*O*-glucoside	86.49%	(Paun et al., 2022)
			Delphinidin-3-xyloside	0.26%	
			Cyanidin-3-*O*-arabinoside	6.40%	
			Cyanidin-3-*O*-malonyl-glucoside	2.15%	
			Cyanidin-3-*O*-rutinoside	4.57%	
*Rubus fruticosus* cv. Hull	Maceration • 69.87% ethanol acidified with HCl 0.53% • solid to liquid ratio of 1:19.06 w/v • at 47.68 °C for 17.04 h.	UPLC-QTOF-MS	Cyanidin-3-*O*-glucoside	-	(Wu et al., 2022)
			Cyanidin-3-*O*-(6”-acetylglucoside)		
			Cyanidin-3-*O*-arabinoside		
			Cyanidin-3-*O*-(6”-malonylglucoside)		
			Cyanidin-3-*O*-[6’-*O*-(*p*-coumaroyl)] glucoside		
			pelargonidin 3-O-(6-O-malonyl-beta-D-glucoside)		
			Delphinidin-3-*O-*arabinoside		
			Cyanidin-3-*O-(6”-*succinylglucoside)		
			Delphinidin-3-*O-*rutinodise		
*Rubus fruticosus* cv. Thornfree	Maceration • 1 g sample extracted with methanol acidified with trifluoro acetic acid (TFA), 0.1%) in a ratio of 1:10 (w/v)	HPLC-PDA	Cyanidin-3-*O-*glucoside	32926 mg/100 g DW	(Memete et al., 2023)
			Cyanidin-3-*O*-arabinoside	1740 mg/100 g DW	
			Cyanidin 3-O-(6-O-malonyl-β-D-glucoside)	1283 mg/100 g DW	
			Cyanidin 3-(6”-dioxalylglucoside)	1798 mg/100 g DW	
			Cyanidin-3-*O-*rutinoside	1492 mg/100 g DW	
*Rubus occidentalis*	Maceration • solid-solvent ratio (1:80), extraction time 60 min using methanol 85% acidified 0.5% formic acid.	UHPLC-QTOF-MS/MS.	Cyanidin-3,5-*O-*diglucoside	-	(Chan et al., 2023)
			Cyanidin-3-*O*-galactoside		
			Cyanidin-3-*O*-glucoside		
			Cyanidin-3-*O*-sambubioside		
			Cyanidin-3-*O*-rutinoside		
			Cyanidin-3-*O-*xylosyl-rutinoside		
			Pelargonidin-3-*O*-rutinoside		
*Rubus fruticosus* cv. Shuofeng	UAE • 3 kg frozen Shuofeng blackberry extracted with 70% ethanol (containing 0.1% formic acid by volume) • Temperature at 40°C and 60 Hz for 30min	UPLC-QTOF-MS	Cyanidin-3-*O-*glucoside	26.67%	(Zhao et al., 2023)
			Cyanidin-3-*O*-arabinoside	0.74%	
			Cyanidin-3-*O*-xyloside	5.19%	
			Cyanidin 3-O-(6-O-malonyl-β-D-glucoside)	8.88%	
			Cyanidin 3-(6”-dioxalylglucoside)	7.41%	
			Pelargonidin 3-O-(6-O-malonyl-β-D-glucoside)	23.70%	
*Rubus coreanus* Miquel	UAE-NADES • NADES consisted of choline chloride, malic acid, and 1,4-butanediol (2:1:1; w/w/w) • Extraction 15 min t temperature at 26 °C, • NADESs water content of 23.3% (v/v)	LC-PDA and LC-QTOF-MS	Cyanidin-3-*O*-glucoside	3200 mg/100 g	(Kim et al., 2024)
*Rubus glaucus* Benth	Maceration • 1 g powdered samples extracted using methanol 80% acidified with 0.1% hydrochloric acid. • The mixture was stirred for 2 h at room temperature • Centrifuged for 10 min at 5.000 rpm (10°C).	HPLC DAD/ESI-MS	Cyanidin-3-*O*-rutinoside	-	(Guevara-Terán et al., 2024)
			Cyanidin-3-*O*-glucoside		
			Pelagonidin-3-*O*-glucoside		
			Pelagonidin-3-*O*-rutinoside		
*Rubus fruticosus*	UAE • 2 g samples extracted with 10 mL isopropanol 80 % • sonicated at frequency of 40 KHz for 60 min at 25 °C	UHPLC-HRMS	Cyanidin-3-*O*-arabinoside	13.48 mg/100 g DW	(Kodikara et al., 2024)
			Cyanidin-3-*O*-glucoside	36.84 mg/100 g DW	
			Cyanidin-3-*O*-rutinoside	11.04 mg/100 g DW	
			Delphinidin-3-*O*-glucoside	18.02 mg/100 g DW	
			Malvidin-3-*O*-glucoside	0.009 mg/100 g DW	
			Peonidin-3-*O*-glucoside	0.067 mg/100 g DW	
*Rubus idaeus*			Cyanidin-3-*O*-arabinoside	0.022 mg/100 g DW	
			Cyanidin-3-*O*-glucoside	6.563 mg/100 g DW	
			Cyanidin-3-*O*-rutinoside	31.03 mg/100 g DW	
			Delphinidin-3-*O*-glucoside	0.218 mg/100 g DW	
			Malvin	0.0144 mg/100 g DW	
			Malvidin-3-*O*-glucoside	0.009 mg/100 g DW	
			Peonidin-3-*O*-glucoside	0.117 mg/100 g DW	
*Rubus fruticosus*	UAE • 2 g samples extracted with distilled water Solid - Liquid ratio 1:15 • sonicated 11.75 min at • temperature below 45 °C • Ultrasound Amplitude Applied 90% • Output power 400 W	HPLC - DAD	Cyanidin-3-*O*-glucoside	107.9 mg/100 g FW	(Piasecka et al., 2024)
			Cyanidin-3-*O*-arabinoside	1.4 mg/100 g FW	
			Cyanidin-3-xyloside	8.1 mg/100 g FW	
*Rubus idaeus*			Cyanidin-3-*O*-glucoside	31.6 mg/100 g FW	
			Cyanidin-3-*O*-sophoroside	41.8 mg/100 g FW	
			Cyanidin-3-*O*-rutinoside	19 mg/100 g FW	
			Pelargonidin-3-sophoroside	2.8 mg/100 g FW	
*Rubus spp*.	UAE-DES • extracted with choline chloride and glycerol-based DES (CHGLY). • glycerol and choline chloride were mixed at 1:4.22 molar ratio after which 20 % distilled water was added. • 0.5 g of sample mixed with 15 g of DES • sonicated for 15 min at 25 °C	HPLC Agilent 1260 Series	cyanidin-3-*O*-glucoside	0.119 mg/100 g	(Zannou et al., 2025)
			cyanidin-3-*O*-rutinoside	0.014 mg/100 g	
			pelargonidin-3-*O*-glucoside	0.005 mg/100 g	
			Cyanidin chloride	0.008 mg/100 g	
*Rubus fruticosus* Pomace	MAE • 150 g of blackberry pomace powder was mixed with 2000 mL of solvent ethanol: water ratio (30:70). • The power level was 600 W for 30 min • the extraction temperature varied from 30 to 35 °C and vessel pressure was 300 mbar	HPLC	Cyanidin-3-*O*-rutinoside	2.72 ± 0.05 mg/mL	(Čechovičienė et al., 2025)
			Cyanidin-3-*O*-rutinoside	2.13 ± 0.03 mg/mL	
*Rubus occidentalis* Pomace	UAE-NADES • Solvent choline chloride/citric acid NADES with 15.6% (v/v) water content • Extraction 52.9 min temperature at 65 °C • Ultrasonics power 320 W and frequency 35 kHz	HPLC-DAD	Cyanidin-3-*O*-rutinoside	760 mg/100 g DW	(Krgović et al., 2025)
**Malus**					
*Malus* ‘Royalty Fruits	UAE • Sample extracted with solid–liquid ratio of 1:6 (g/mL), ethanol and formic acid contents of 70% and 0.4% as solvent • temperature at 20 °C and 40 min • ultrasonic power of 300 W	HPLC-DAD	Cyanidin-3-*O*-galactoside	-	(Liu et al., 2022)
*Malus × domestica Borkh*	UAE • apple pieces (∼400.0 mg each) were rapidly powdered • extracted by ultrasound-assisted maceration for 1 h using methanol-formic acid solution (19:1) v/v	LC-MS/MS	Cyanidin-3-*O*-pentoside	-	(Faramarzi et al., 2015)
			Cyanidin-3-*O*-hexoside		
			Cyanidin-3-*O*-galactoside		
*Malus domestica* Borkh	UAE • 50 mg powders of Samples were extracted during 30 min in an ultrasonic bath with 1.5 mL of methanol (MeOH) containing 5% acetic acid (v/v)	UPLC–Q-TOF-MS	Cyanidin-3-*O*-galactoside	0.237-30.947 mg/100 g FW	(Bouillon et al., 2024)
White-fleshed apples	Maceration • apple flesh (0.2 g) and peel (0.1 g) extracted with of methanol/Milli-Q water/acetic acid (79.9:20:0.1, v/v/v) as	UPLC-MS/MS	Cyanidin-3-*O*-galactoside	0.034 mg/100 g FW	(Bars-Cortina et al., 2017)
White-fleshed apples peels			Cyanidin-3-*O*-galactoside	31.5 mg/100 g FW	
Red-fleshed apples			Cyanidin-3-*O*-galactoside	4.54 mg/100 g FW	
Red-fleshed apples peels			Delphinidin rhamnoside	0.81 mg/100 g FW	
			cyanidin-3-*O*-galactoside	16.7 mg/100 g FW	
Traditional apple CRVENKA cultivar peels	UAE • 3 g Apple peel extracted with 22.5 ml of 80% methanol the solution was vortexed • extracted with the help of ultrasonic bath for 15 min. The residue was then extracted one more time with the same procedure using 10 ml of 80% methanol in water	RP-HPLC-PDA	cyanidin-3-*O*-galactoside	19.66 mg/100 g FW	(Jakobek et al., 2021)
**Pyrus**					
*Pyrus communis* peels	UAE • sample extracted using solvent and sample ratio 1:30 g/ml with ethanol 57% containing trifluoroacetic acid 3% • sonicated for 11 min with 162 W of ultrasonic power temperature at 71 °C,	UPLC-Triple-TOF/MS	Cyanidin-3-*O*-galactoside	-	(Belwal et al., 2019)
			Cyanidin-3,5-*O*-diglucoside		
			Cyanidin-3-*O*-rutinoside		
			Cyanidin-3-*O*-arabinoside		
			Delphinidin 3-*O*-glucoside		
			Petunidin-3-*O*-galactoside		
			Petunidin-3-*O*-galactoside		
**Fragaria**					
*Fragaria x ananassa* Duch	UAE • 20 g sampel extracted with 20 mL Acetone • Sonicated 10 min at 20 C, with frequency of 35 kHz. • Filter the mixture using a Büchner funnel and Whatman No. 1 filter paper. • The residue on the filter paper is re-extracted twice using 10 ml of acetone 70%	HPLC-UV/VIS-PDA	Cyanidin-3-*O*-rutinoside	0.366 mg/100 g FW	(Kovačević et al., 2015)
			Pelargonidin-3-*O-*glucoside	15.424 mg/100 g FW	
			Pelargonidin-3-*O*-rutinoside	0.088 mg/100 g FW	
			Cyanidin-3-*O*-glucoside	0.711 mg/100 g FW	
*Fragaria* spp.	UAE • 5 g sample extracted with 10 mL solvent 0.20% HCl in methanol. • Sonicated 10 min in ultrasound bath at 20 C using as extracting • the extraction procedure was repeated four times	RPLC-DAD	Pelargonidin-3-*O*-glucoside	4.91 mg/100 g	(Canuto et al., 2016)
			Pelargonidin-3-*O*-rutinoside	0.217 mg/100 g	
			Cyanidin-3-*O*-glucoside	2.42 mg/100 g	
*Fragaria × Ananassa* cv. Romina	Maceration • 50 g of strawberries homogenized in 100 mL of methanol containing 0.1% HCl • stirred overnight and subsequently filtered through a Büchner funnel under vacuum.	RPHPLC- DAD	Cyanidin-3-*O*-glucoside	398 mg/100 g DW	(Forbes-Hernández et al., 2017)
			Pelargonidin-3,5-diglucoside	155 mg/100 g DW	
			Pelargonidin-3-*O*-glucoside	26676 mg/100 g DW	
			Pelargonidin-3-malonylglucoside	5950 mg/100 g DW	
			Pelargonidin-3-acetylglucoside	87 mg/100 g DW	
*Fragaria x ananassa* Duch cv. Seolhyang	UAE • Sample extracted 3 times for 1 h each with 80% methanol containing 0.1% HCl by a sonication extractor • The extract was fil tered with Whatman No. 2 Filter paper	HPLC-PDA	Cyanidin-3-*O*-glucoside	3.61g/100 g DW	(Park et al., 2020)
			Pelargonidin-3-*O*-glucoside	55.1 g/100 g DW	
			Pelargonidin-3-*O*-rutinoside	1.65 /100 g DW	
*Fragaria spp.*	UAE • 2 g of fruits or jam extracted with 10 mL of ethanol 70% acidified with HCl (1.5%) • sonication at a frequency of 40 KHz for 60 min at 25 °C	HPLC-MS/MS	Cyanidin-3-*O*-glucoside	1.386 mg/100 g FW	(Mustafa et al., 2022)
			Pelargonidin-3-*O*-rutinoside	0.543 mg/100g FW	
			Pelargonidin-3-*O*-glucoside	18.2 mg/100 g FW	
*Fragaria × ananassa* Duch.	UAE-NADES • Sample extracted with liquid-to-solid ratio of 33 mL/g using solvent Choline chloride-citric acid (1:1) • Sonication 19 min at of 61 °C with ultrasonic power of 318 W	UPLC-Triple-TOF/MS	Delphinidin-3-*O*-glucoside	3%	(Huang et al., 2024)
			Cyanidin-3-*O*-glucoside	12%	
			Cyanidin-3-*O*-rutinoside	7%	
			Pelargonidin-3-*O*-glucoside	65%	
			Pelargonidin-3-*O*-rutinoside	8%	
			Malvidin-3-*O*-glucoside	5%	
*Fragaria × ananassa* cv. Benihoppe	MAE • 1 gram of powder was mixed with 10 mL 70 % ethanol (with 1 % formic acid) • processed with microwave 350 W for 15 s	UHPLC-QTOF-MS	Pelargonidin3-*O*-glucoside	40.761 mg/100 g DW	(Zeng et al., 2025)
			Cyanidin-3-*O*-glucoside	2.6214 mg/100 g DW	
**Prunus**					
*Prunus avium*	Maceration • 10mL/g solvent/solid sample ratio extracted for 90 min at 37°C using 100% ethanol containing 0.1%v/v 12N HCl)	UPLC-DAD	Peonidin-3-*O*-rutinoside	15 %	(Blackhall et al., 2018)
			Cyanidin-3-*O*- rutinoside	80 %	
			Pelargonidin-3-*O*-rutinoside	5%	
*Prunus avium*	Maceration • 12 mL/g cherry powder extracted using methanol 72% acidified with 1% formic acid at 55 °C • samples were centrifuged at 9,500 g for 10 min at 4 °C	HPLC-ESI-MS/MS	Cyanidin-3-*O*-arabinoside	1.393 mg/100 g DW	(Iglesias-Carres et al., 2019)
			Cyanidin 3-(6-p-caffeoyl) glucoside	0.778 mg/100 g DW	
			Cyanidin-3-*O*-glucose	21.383 mg/100 g DW	
			Cyanidin-3-*O*-rutinoside	94.291 mg/100 g DW	
			Delphinidin-3*-O*-rutinoside	0.014 mg/100 g DW	
			Delphinidin-3-*O*-(6-p-coumaroyl) glucoside	9.761 mg/100 g DW	
			Malvidin-3-*O*-glucoside	0.036 mg/100 g DW	
			Pelargonidin-3-*O*-glucoside	0.781 mg/100 g DW	
			Peonidin-3-*O*-rutinoside	3.297 mg/100 g DW	
*Prunus cerasus* peels	MAE • 500 W of microwave power, • 90 seconds of irradiation time • 80% ethanol	HPLC-DAD	cyanidin-3-*O*-glucoside	1230 mg/100 g FW	(Kurtulbaş Şahin et al., 2021)
*Prunus nepalensis*	UAE • 300 W ultrasonic power • 40% ethanol containing 2% hydrochloric acid • Solid- liquid ratio of 1:20 g/ml • 30 min ultrasonic time • Temperature at 60°C	UHPLC–ESI–MS/MS	cyanidin-3-*O*-glucoside	231.594 mg/100 g	(Ma et al., 2022)
			Petunidin-3-*O*-glucoside	2779.129 mg/100 g	
*Prunus spinosa*	Reflux • Fresh fruits (1008.0g) poured with a small amount of heated methanol water (75:25, v/v), ground in a blender • refluxed three times with methanol-water (75:25, v/v; 1.5 L and 20 min each)	LC-PDA-ESI-MS^3^	Cyanidin-3-*O*-rutinoside	139 mg/100 g DW	(Magiera et al., 2022)
			Cyanidin-3-*O*-glucoside	196 mg/100 g DW	
			Peonidin-3-*O*-glucoside	139 mg/100 g DW	
*Prunus cerasus*	UAE • 0.4 g of fresh/dried sample • 10 mL of double distilled water (DDW) • 25 % amplitude, 200 W power, 20 kHz frequency • 9 min extraction time, • Temperature 20 ± 2 °C	UHPLC-HRMS	cyanidin-3-*O*-rutinoside	9.81 mg/L	(Ebrahimi et al., 2025)
*Prunus spp*	UAE • 2 g samples extracted with 10 mL isopropanol 80 % • sonicated at frequency of 40 KHz for 60 min at 25 °C	UHPLC-HRMS	Cyanidin-3-*O*-arabinoside	0.2349 mg/100 g DW	(Kodikara et al., 2024)
			Cyanidin-3-*O*-glucoside	6.6060 mg/100 g DW	
			Cyanidin-3-*O*-rutinoside	31.56 mg/100 g DW	
			Delphinidin-3-*O*-glucoside	0.8624 mg/100 g DW	
			Malvidin-3-*O*-glucoside	0.0054 mg/100 g DW	
			Peonidin-3-*O*-glucoside	0.2258 mg/100 g DW	

FW(Fresh Weight); DW(Dry Weight).

**Fig. 4:**
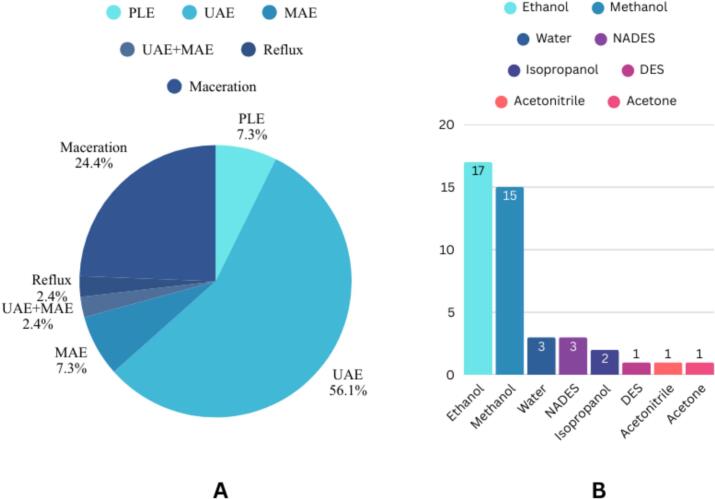
Distribution of extraction techniques and solvents used for anthocyanin-rich extract preparation from Rosacea fruits. (A) Proportion of extraction methods reported in the literature, highlighting ultrasound-assisted extraction (UAE) as the most frequently applied technique. (B) Frequency of solvents employed, showing ethanol and methanol as the predominant extraction solvents compared to other conventional and alternative solvent systems.

Based on the data presented in Table 1, UAE was identified as the most frequently applied method for anthocyanin extraction from fruits of the Rosaceae genus. This finding is consistent with previous reports highlighting the advantages of UAE in extracting anthocyanins from plant matrices. In contrast, the choice of solvents varied considerably, including acetone, methanol, ethanol, water, acetonitrile, and isopropanol, as well as green solvents such as DES and NADES. A summary of the extraction methods and types of organic solvents employed is presented in Fig. 4, where ethanol emerged as the most frequently used solvent, followed by methanol. ethanol was identified as the most frequently used solvent, followed by methanol. Ethanol is a polar solvent that is highly effective for extracting bioactive compounds due to its broad solubility range as a neutral solvent, enabling the extraction of diverse compounds. Technically, ethanol is relatively safe and economical, and its rapid evaporation after extraction facilitates the concentration process. These characteristics make ethanol widely employed for the extraction of bioactive substances, particularly those with high antioxidant activity, and it is extensively applied in the food and beverage industries (Azwanida, 2015). In contrast, methanol, although possessing several advantages such as low cost and high extraction efficiency, also presents notable limitations. Methanol is highly volatile and flammable, thereby posing safety risks, while its environmental toxicity renders it unsuitable for the implementation of green chemistry principles. Therefore, the use of methanol as a solvent must be carefully aligned with regulatory requirements and appropriate safety management practices (Lee et al., 2024). Furthermore, the concentration of organic solvents varied according to the sample type and the optimization results reported in previous studies. In addition, to enhance the extraction yield of anthocyanins, organic solvents were commonly acidified with weak organic acids such as citric acid, formic acid, trifluoroacetic acid, and acetic acid, or with strong acids like HCl at low concentrations.

**Fig. 5:**
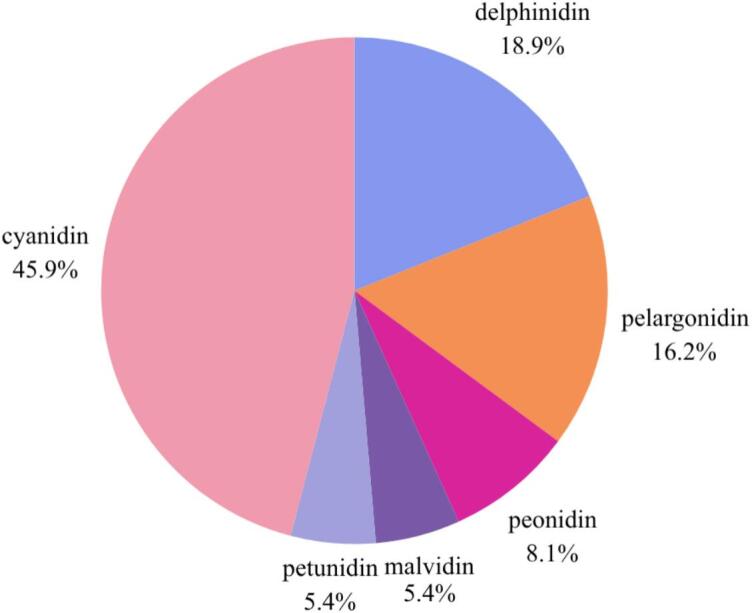
Distribution of anthocyanidins identified in Rosaceae fruits. Cyanidin derivatives represent the most dominant group (45.9%), followed by delphinidin (18.9%), pelargonidin (16.2%), peonidin (8.1%), malvidin (5.4%), and petunidin (5.4%).

Furthermore, a total of 37 anthocyanin compounds were identified from various fruits belonging to the Rosaceae family. The structural diversity of anthocyanins is illustrated in Fig. S1. Cyanidin was the most dominant, with 17 distinct compounds, followed by delphinidin (seven), pelargonidin (six), peonidin (three), and both malvidin and petunidin, each represented by two distinct compounds. The distribution of anthocyanidins in Rosaceae fruits is presented in Fig. 5. Cyanidin-based anthocyanins were the most frequently identified across the surveyed fruits, indicating that cyanidin serves as the predominant structural backbone within this family, particularly cyanidin-3-glucoside, which is present in nearly all fruits of the Rosaceae family. This observation is consistent with previous reports, which noted that cyanidin-based anthocyanins are the most prevalent, followed by delphinidin and pelargonidin (Roy & Rhim, 2021). The prevalence of cyanidin, delphinidin, and pelargonidin can be attributed to their direct biosynthetic origin from naringenin via the action of F3H enzym, which converts naringenin into dihydroquercetin, dihydrokaempferol, and dihydromyricetin, respectively. In contrast, peonidin, petunidin, and malvidin are formed through methylation of hydroxyl groups on cyanidin, delphinidin, and pelargonidin, catalyzed by specific O-methyltransferases. This biosynthetic divergence highlights the enzymatic modulation that contributes to anthocyanin diversity and may influence their functional properties in plant physiology and human health applications (Sunil & Shetty, 2022).

Further analysis revealed that fruits from the genus *Rubus*, including blackberry, raspberry, and black raspberry, exhibit anthocyanin profiles predominantly composed of cyanidin derivatives, with 17 distinct compounds identified. Additionally, four anthocyanins derived from delphinidin, four from pelargonidin, two from malvidin, and one from peonidin were also detected. Notably, no petunidin-derived anthocyanins were identified in *Rubus*, suggesting that the biosynthetic pathway leading to petunidin may be inactive or less prominent compared to those responsible for the synthesis of cyanidin, delphinidin, pelargonidin, peonidin, and malvidin. Simultaneously, Fruits from the genus *Malus*, such as apples, exhibited the presence of seven anthocyanin compounds derived from three major anthocyanidin classes. Among these, five were cyanidin derivatives and one anthocyanin each from delphinidin and peonidin was identified. This distribution suggests that anthocyanin biosynthesis in *Malus* tends to follow a relatively simple pattern, with a clear predominance of the cyanidin biosynthetic pathway. The limited diversity of anthocyanidin classes may reflect constrained enzymatic activity or substrate specificity within the flavonoid biosynthetic network in this genus.

On the other hand, fruits from the genus *Pyrus* exhibited a more diverse anthocyanin profile. A total of five anthocyanins were derived from the cyanidin class, indicating a dominance pattern similar to that observed in *Rubus* and *Malus*. However, *Pyrus* also contained anthocyanins from the peonidin, petunidin, delphinidin, and pelargonidin classes. In contrast, fruits from the genus *Fragaria*, such as strawberries, exhibited a distinctive anthocyanin distribution pattern compared to other genera within the Rosaceae family. Pelargonidin-based anthocyanins were the most prevalent, with five distinct compounds identified. This observation diverges from the typical anthocyanin profile of other Rosaceae fruits, in which cyanidin derivatives are generally the most abundant. The predominance of pelargonidin in *Fragaria* reflects a divergent biosynthetic pathway, characterized by relatively low activity of F3′H and F3′5′H. As a result, anthocyanin biosynthesis in this genus is directed toward pelargonidin rather than cyanidin or delphinidin. Only two cyanidin-derived anthocyanins were identified in *Fragaria*, along with one compound each from malvidin and delphinidin. This pattern suggests that *Fragaria* holds potential as a natural source of pelargonidin-type anthocyanins, which may offer distinct functional and pharmacological properties compared to cyanidin-dominant profiles observed in other Rosaceae fruits.

Lastly, fruits from the genus *Prunus*, including cherries and plums, exhibited a relatively balanced anthocyanin profile. Studies have demonstrated that this genus contains all six major anthocyanidin types. The presence of multiple anthocyanidin types further indicates a complex regulation of gene expression and enzyme specificity within the flavonoid biosynthetic pathway in this genus, which may vary across cultivars, developmental stages, and environmental conditions. Anthocyanins derived from cyanidin, delphinidin, and pelargonidin are the most frequently identified, indicating the presence of active biosynthetic pathways involving hydroxylation at the 3′ and 5′ positions of the flavonoid B-ring, catalyzed by the enzymes F3′H and F3′5′H. In contrast, anthocyanins such as peonidin, petunidin, and malvidin are formed through methylation of hydroxyl groups on cyanidin, delphinidin, and pelargonidin, a reaction catalyzed by specific O-methyltransferases.

In terms of sugar moieties, the most identified groups in anthocyanin structures from Rosaceae fruits belong to monosaccharides and disaccharides. Among the monosaccharide substituents, glucoside, galactoside, and arabinoside were the most frequently detected. Meanwhile, rutinoside, sambubioside, and sophoroside represented the dominant disaccharide groups. In addition, several anthocyanins underwent acylation, resulting in acylated forms such as malonyl-glucoside, acetyl-glucoside, and succinyl-glucoside. These findings indicate that the enzymes UFGT and AAT play a pivotal role in anthocyanin modification in Rosaceae fruits through glycosylation and acylation processes. Such structural modifications are crucial for enhancing the stability, biological activity, and resistance of anthocyanins to degradation within plant tissues (Cappellini et al., 2021). The remarkable diversity of anthocyanin compounds found in Rosaceae fruits reflects a high degree of biochemical variation and opens promising avenues for further exploration in the development of functional products based on natural compounds.

The remarkable diversity of anthocyanin compounds found in Rosaceae fruits reflects a high degree of biochemical variation and opens promising avenues for further exploration in the development of functional products based on natural compounds. The identification of anthocyanins across Rosaceae cultivars remains limited, although the data in the table indicate that several cultivars generally reveal similar qualitative profiles. Nevertheless, literature published over the past decade shows that studies directly comparing the influence of environmental conditions and cultivar variation on anthocyanin composition are still scarce. This limitation highlights a research gap that future investigations could address by focusing on the combined effects of environmental variability and cultivar diversity.

## 8 Bioactivitiy of anthocyanins-rich extract from Rosacea fruits

### 8.1 Antioxidant

Lipid peroxidation is considered one of the principal molecular mechanisms involved in oxidative damage to cellular structures and in the toxicity processes that lead to cell death, which can trigger various diseases. Therefore, the inhibition or reduction of lipid peroxidation activity is crucial in the prevention of such diseases, making the role of antioxidants highly significant. This process can be inhibited through enzymatic reactions or by scavenging free radicals using antioxidant compounds such as anthocyanins (Forbes-Hernández et al., 2017). Anthocyanins are antioxidant compounds that function to prevent or inhibit oxidative stress through the donation of H atoms or single electron transfer. The structure of anthocyanins, particularly the orientation of the ring and the sugar moieties bound to the B-ring, plays a crucial role to determining the level of their antioxidant activity (Tena et al., 2020).

Numerous studies have demonstrated that the consumption of anthocyanin-rich fruits, particularly those from the Rosaceae family, exerts beneficial effects on human health, primarily through the mitigation of oxidative stress. According to a study in vivo study by Shen et al. (2022) revealed that anthocyanin extracts from Xinjiang wild cherry plum (*Prunus divaricata*) enhanced plasma antioxidant capacity by upregulating antioxidant enzyme activity and reducing oxidative stress levels. These findings underscore the role of anthocyanins in modulating oxidative stress. The consumption of anthocyanins derived from raspberry fruit also significantly enhances the activity of the antioxidant enzyme superoxide dismutase (SOD) and markedly reduces malondialdehyde (MDA) levels, indicating an improvement in antioxidant status. This effect was demonstrated through an in vivo study conducted by (Wu, Gao, et al., 2018; Wu, Yang, et al., 2018), C57BL/6 mice were administered low-fat, high-fat, and high-fat diets supplemented with raspberry anthocyanins (RA) at a dose of 200 mg/kg for 12 weeks to evaluate their impact on antioxidant activity. The results confirmed that RA supplementation effectively improved antioxidant enzyme activity and mitigated lipid peroxidation, highlighting the potential of raspberry anthocyanins in enhancing systemic oxidative defense mechanisms. Similarly, a study conducted by Park et al. (2020) revealed that anthocyanin extract from Seolhyang strawberry cultivar exhibited a reduction value (RC₅₀) of 0.66 ± 0.15 mg/mL. The RC₅₀ indicates the concentration required to achieve a 50% reduction in free radicals.

### 8.2 Anti-inflammatory

Inflammation is a fundamental biological response that plays a critical role in defending the body against infection and injury. The primary goal of the inflammatory response is to eliminate the harmful agent, remove damaged cells, and initiate tissue repair. Clinically, inflammation is manifested by cardinal signs, including redness, swelling, heat, pain, and functional impairment (Kowalczyk et al., 2024). However, when inflammation becomes persistent, it may progress into a chronic state, posing significant health risks. Chronic inflammation can occur when the immune system fails to effectively neutralize harmful stimuli or terminate the inflammatory response. Unlike acute inflammation, which is a necessary and beneficial reaction to infection or injury, chronic inflammation contributes to the development and progression of various diseases, including diabetes, cardiovascular disorders, neurodegenerative conditions, and cancer (Nigam et al., 2023).

Recent evidence indicates that regular consumption of fruits from the Rosaceae family may contribute positively to the modulation of inflammatory pathways. According to a study by Bialasiewicz et al. (2018), regular consumption of 0.5 kg of sour cherries over 30 days significantly reduced oxidative activity in the blood, specifically rLBCL and fMLP-LBCL, in healthy individuals. This reduction is attributed to the bioactive constituents of sour cherries, particularly anthocyanins, which inhibit the formation of reactive oxygen species (ROS), both basal and agonist-induced, by circulating phagocytes. A comprehensive study conducted by Harlan et al. (2020) also demonstrated that anthocyanins derived from tart cherry (*Prunus cerasus*) exert significant anti-inflammatory effects, primarily through modulation of the NFκB signaling pathway. Using 3T3-L1 adipocytes treated with tart cherry extract containing 18–36 μg/mL of anthocyanins, the researchers evaluated both individual and combined effects of tart cherry anthocyanins (TCA) and delta-tocotrienol (DT3) on inflammatory responses. The study confirmed that neither TCA nor DT3, alone or in combination, induced cytotoxicity, thereby preserving adipocyte viability throughout the treatment. Protein-level analysis revealed that TCA markedly reduced the expression of key inflammatory markers, including interleukin-6 (IL-6) and p-65 (a subunit of NFκB), in the culture media. Additionally, downstream targets of NFκB, macrophage inflammatory protein 2 (Mip2) and cyclooxygenase-2 (Cox2), were significantly downregulated (p ≤ 0.05) following treatment. These findings affirm the potent anti-inflammatory properties of TCA in adipocyte models and underscore the need for further investigation into the integrated roles of DT3 and TCA, particularly within the JNK and MAPK signaling cascades.

In vivo studies have demonstrated that anthocyanin-rich extracts derived from these fruits significantly attenuate inflammation, primarily through the downregulation of key pro-inflammatory mediators. A study conducted by Lim et al. (2020) investigated the effects of sustained consumption of black raspberry (BR) extract on hypercholesterolemia and hepatic inflammation induced by excessive choline intake in rats maintained on a high-fat diet. Excessive choline intake has been implicated in the development of hypercholesterolemia and liver inflammation, partly through activation of the nuclear factor-κB (NF-κB) signaling pathway. Regular administration of BR extract significantly downregulated the mRNA expression of pro-inflammatory genes, including *NF-κB*, interleukin-1β (*IL-1β*), *IL-6*, and cyclooxygenase-2 (*COX-2*), as well as the protein expression of *NF-κB* and *COX-2* in hepatic tissues. These molecular changes were accompanied by reductions in cecal trimethylamine (TMA) and serum trimethylamine-N-oxide (TMAO) levels, suggesting that consistent intake of BR extract may attenuate choline-induced hypercholesterolemia and hepatic inflammation under high-fat dietary conditions.

In addition, Krikorian et al. (2023) revealed that anthocyanin supplementation derived from strawberries plays a significant role in reducing dementia risk, particularly when introduced during midlife. In a controlled clinical trial, daily intake of 13 grams of whole strawberry powder significantly alleviated memory impairments and depressive symptoms in overweight middle-aged individuals. The anti-inflammatory properties of anthocyanins were identified as the primary mechanism underlying these neurocognitive benefits, reinforcing the potential of strawberry supplementation as a preventive strategy against dementia when implemented during the transitional stages of adulthood. The neuroprotective effects observed in strawberry fruits are likely attributable to their bioactive constituents, particularly anthocyanins and flavonoids, which are known to modulate oxidative stress and inflammatory signaling pathways implicated in the pathogenesis of neurodegenerative disorders.

### 8.3 Anti-obesity

Obesity represents a major global public health challenge and currently ranks as the fifth leading cause of mortality worldwide. It arises from a fundamental imbalance between energy intake and expenditure, most commonly driven by excessive consumption of high-fat, high-sugar diets combined with insufficient physical activity (Ibrahim et al., 2021). This imbalance is compounded by increasingly sedentary lifestyles, which in severe cases may result in impaired autonomous mobility. The health implications of obesity are profound it is a major contributor to reduced life expectancy and a significant risk factor for premature mortality. Moreover, obesity is closely associated with a wide spectrum of chronic diseases, including type 2 diabetes mellitus, hypertension, cardiovascular disorders, and various forms of cancer. Individuals with obesity exhibit systemic inflammation driven by dysfunctional adipose tissue, which secretes pro-inflammatory mediators in response to excessive fat accumulation in the bloodstream (Ngamsamer et al., 2022).

Acknowledging the health risks associated with obesity, it is essential to implement preventive measures to mitigate its progression and associated complications. One such approach involves the consumption of fruits from the *Rosaceae* family, which are rich in anthocyanins. Beyond their antioxidant properties, anthocyanins have also demonstrated therapeutic potential in mitigating obesity-related metabolic disorders. According to a study conducted by (Wu, Gao, et al., 2018; Wu, Yang, et al., 2018) reported anthocyanins derived from raspberry and blackberry exhibit anti-obesity effects. In their study, C57BL/6 mice were assigned to receive either a low-fat diet, a high-fat diet, or a high-fat diet supplemented with Raspberry anthocyanins and Blackberry anthocyanins at a dose of 200 mg/kg for 12 weeks. Raspberry anthocyanins supplementation resulted in a 63.7% reduction in body weight gain, while blackberry anthocyanins inhibited weight gain by 40.5% compared to the unsupplemented high-fat diet group. These findings suggest that regular consumption of anthocyanins derived from raspberry and blackberry may offer protective effects against diet-induced adiposity and support their role in obesity prevention strategies.

In addition, tart cherry (*Prunus cerasus*) also emerged as a promising agent with anti-obesity and anti-inflammatory potential. Study by Cocci et al. (2021) demonstrated that bioactive compounds derived from tart cherry can attenuate adipogenesis in rats fed a high-fat diet (HFD) by directly modulating visceral adipose tissue. The study revealed that tart cherry anthocyanins influence the transcriptional regulation of cannabinoid receptor 1 (CB1), peroxisome proliferator-activated receptor gamma (PPARγ), and transient receptor potential vanilloid (TRPV) channels. A study conducted by (Moruzzi et al., 2021) investigated the effects of a tart cherry-enriched diet on inflammation in rats fed a high-fat, hypercaloric diet. The rats were fed ad libitum for 17 weeks with dietary supplementation of tart cherry seed powder (DS) or a combination of seed powder and tart cherry juice containing 1 mg of anthocyanins (DJS). The results indicated that the tart cherry-enriched diet did not significantly alter visceral fat accumulation; however, it led to a reduction in inflammatory markers in both retroperitoneal and perigonadal adipose tissues. These findings suggest that tart cherry supplementation, when combined with a healthy lifestyle, may beneficially modulate adipose tissue metabolism and help prevent obesity-related organ damage. Complementary results were reported by Moruzzi et al. (2021), who investigated the anti-inflammatory effects of a tart cherry-enriched diet in rats subjected to a high-fat, hypercaloric regimen. Over a 17-week period, animals received dietary supplementation with tart cherry seed powder (DS) or a combination of seed powder and tart cherry juice containing 1 mg of anthocyanins (DJS). While visceral fat accumulation remained largely unchanged, both DS and DJS treatments led to a significant reduction in inflammatory markers within retroperitoneal and perigonadal adipose tissues. These outcomes suggest that tart cherry supplementation, particularly when integrated into a balanced lifestyle, may beneficially modulate adipose tissue metabolism and mitigate obesity-related inflammatory damage.

### 8.4 Antidiabetic

Anthocyanin-rich fruits from the Rosaceae family have demonstrated promising potential as antidiabetic agents, owing to their ability to modulate key metabolic enzymes and improve glycemic control in both in vitro and in vivo models. A study conducted by Dodier et al. (2021) revealed the effects of tart cherry (*Prunus cerasus*) juice on bone metabolism in older postmenopausal women. Twice-daily consumption of tart cherry juice was found to attenuate bone resorption, providing a foundation for future long-term clinical trials aimed at evaluating skeletal responses to anthocyanin-rich whole-fruit interventions. These outcomes underscore the broader therapeutic potential of Rosaceae fruits in modulating vascular, metabolic, and skeletal health. Furthermore, a study conducted by Magiera et al. (2022) also demonstrated that the consumption of fresh fruits of *Prunus spinosa* exerts inhibitory effects on glycolytic enzymes, particularly α-glucosidase, a key therapeutic target in antidiabetic treatment. This potent enzyme inhibition is attributed to the high polyphenolic content of the extract, which is presumed to exert its biological effects through additive and synergistic interactions among its bioactive constituents. Not only *Prunus spinosa*, but anthocyanin extracts derived from *Prunus domestica* peels have also demonstrated promising potential in modulating hepatic and renal enzyme activity, as well as contributing to glycemic regulation during sustained administration.

In addition to fruits from the *Prunus* genus, strawberries and blackberries have also been reported to exhibit antidiabetic activity. (Park et al., 2020) demonstrated that anthocyanin extract from the Seolhyang strawberry cultivar exhibited α-glucosidase inhibitory activity, with an IC₅₀ value of 0.74 ± 0.01 mg/mL. α-Glucosidase inhibitors (AGIs) suppress the activity of the α-glucosidase enzyme, thereby delaying glucose absorption and contributing to postprandial glycemic control. Furthermore, purified blackberry anthocyanin extract also has the anti-hyperglycemic activity. As reported by Wu et al. (2022), was significantly more effective than that of crude anthocyanin extract and blackberry fruit slurry extract. The IC₅₀ value of purified blackberry anthocyanin extract in the α-amylase inhibitory assay was 0.10 ± 0.01 mg/mL, while its α-glucosidase inhibitory activity yielded an IC₅₀ value of 0.06 ± 0.01 mg/mL. These findings highlight the importance of anthocyanin extraction and purification techniques in enhancing bioactivity, particularly in modulating carbohydrate metabolism and mitigating hyperglycemia. In an in vivo study conducted by Omran et al. (2025), rats were orally administered the extract at a dose of 100 μg/kg/day over durations ranging from one to four weeks. The intervention led to a significant reduction in glutamic oxaloacetic transaminase (GOT) levels, which remained within the normal physiological range throughout the study. Similarly, glutamic pyruvic transaminase (GPT) levels were consistently reduced and maintained within typical limits over the four-week period, indicating improved hepatic function and metabolic stability.

### 8.5 Anticancer

Cancer has become a major global public health challenge, with over 52.900 individuals diagnosed and more than 27,000 deaths recorded daily due to the disease. It is characterized by abnormal and uncontrolled cell growth that can result in tumor formation and metastases, which refers to the spread of cancer cells to other parts of the body (Kowalczyk et al., 2024). To date, conventional therapies such as chemotherapy and radiation remain widely used in cancer treatment; however, they often impose substantial physical and psychological burdens on patients. Consequently, many researchers have explored natural compounds as alternative anticancer agents, among which anthocyanins have gained considerable attention. Anthocyanins from Rosaceae have emerged as promising candidates due to their potent antioxidant activity and their ability to induce apoptosis, inhibit cancer cell proliferation, suppress angiogenesis, and reduce metastasis (Kowalczyk et al., 2024).

Beyond anticancer effects, Desai et al. (2021) provided the first clinical evidence that seven-day supplementation with Montmorency tart cherry juice (*Prunus cerasus*) significantly improved 24-hour ambulatory blood pressure, reduced fasting glucose and total cholesterol levels, lowered the total cholesterol-to-HDL ratio, and decreased resting respiratory exchange ratio in individuals with metabolic syndrome (MetS). These findings underscore the clinical potential of Montmorency tart cherry juice as a functional nutritional intervention to prevent further cardio-metabolic dysregulation in at-risk populations. Further evidence of the anticancer potential of Rosaceae fruits was reported by Omran et al. (2025), who demonstrated that *Prunus domestica* peel extracts exhibited enhanced cytotoxicity against MCF-7 breast cancer cells at higher concentrations. The anthocyanin-rich extract yielded an IC₅₀ value of 34.92 μg/mL, indicating strong anticancer activity. Additionally, anthocyanins extracted from *Malus* 'Royalty' fruits also exhibited significant antitumor effects against the gastric cancer cell line BGC-803, as reported by Yixin Liu et al. (2022). In vitro cell viability assays using the CCK-8 method revealed a dose-dependent inhibition of tumor cell proliferation, with an IC₅₀ value of 105.5 μg/mL, indicating notable cytotoxic activity.

### 8.6 Antibacterial

Recent studies have highlighted the therapeutic potential of anthocyanin-rich extracts in mitigating bacterial contamination in food. These natural pigments, sourced from a variety of plants, possess significant antibacterial properties, effectively inhibiting the proliferation of multiple bacterial strains (Jeyaraj et al., 2021). Researchers have demonstrated the antibacterial properties of anthocyanins through various mechanisms, including the destruction of bacterial cells and the inhibition of extracellular enzyme activity. Anthocyanins are also known to bind to bacterial DNA, potentially interfering with replication, transcription, and gene expression, ultimately leading to cell death. Furthermore, these compounds can compromise the integrity of bacterial cell walls and membranes, resulting in protein leakage, disruption of protein synthesis, and degradation of essential bacterial proteins. Collectively, these effects significantly impair the metabolism and growth of *Escherichia coli* and other microbial strains (Ikhsan et al., 2025; Jeyaraj et al., 2021).

Fruits belonging to the genera *Rubus*, *Prunus*, and *Malus* have demonstrated notable antibacterial properties, which are hypothesized to be primarily attributed to their anthocyanin content. This hypothesis is supported by several empirical studies. For instance, Barba-Ostria, Carrera-Pacheco, et al. (2024) and Barba-Ostria, Gonzalez-Pastor, et al. (2024) reported that extracts derived from *Rubus glaucus* exhibited pronounced antibacterial efficacy against Gram-positive bacteria, with minimum inhibitory concentrations (MICs) as low as 1 mg/mL. The Gram-positive strains evaluated included *Staphylococcus aureus* ATCC 25923, *Enterococcus faecalis* ATCC 29212, and *Listeria monocytogenes* ATCC 13932. In contrast, the same extracts showed comparatively weaker activity against Gram-negative bacteria, such as *Pseudomonas aeruginosa* ATCC 27853, *Salmonella typhimurium* ATCC 14028, *Burkholderia cepacia* ATCC 25416, and *Escherichia coli* ATCC 25922, with MIC values ranging from 8 to 12 mg/mL. These findings showed that anthocyanins exhibit greater efficacy against Gram-positive bacteria, likely due to their simpler cell wall architecture. Conversely, the outer membrane of Gram-negative bacteria serves as a selective barrier, impeding the penetration and diminishing the bioactivity of these compounds.

Subsequently, Veličković et al. (2021) also reported that ethanol extracts derived from Himalayan blackberry (*Rubus discolor)* exhibited notable antibacterial activity. The extracts were evaluated against a range of bacterial strains, including *Listeria monocytogenes* NCTC 7973, *Micrococcus flavus* ATCC 10240, *Escherichia coli* ATCC 35210, *Pseudomonas aeruginosa* IBRS P001, and *Salmonella typhimurium*. The results indicated that the samples exhibited greater efficacy against Gram-positive bacteria compared to Gram-negative strains, consistent with previous findings. The minimum inhibitory concentration (MIC) and minimum bactericidal concentration (MBC) values ranged from 1.2 mg/mL to 5.0 mg/mL, suggesting moderate to strong antibacterial potential. Similarly, (Wu et al., 2022) reported that purified blackberry anthocyanin extract (BA-PAE) demonstrated significantly greater antimicrobial efficacy compared to both crude anthocyanin extract (BA-CAE) and blackberry fruit slurry extract (BA-FSE). The crystal violet staining assay was employed to quantify the influence of these extracts on *Listeria monocytogenes* biofilm formation. At a concentration of 2.0 mg/mL, BA-PAE achieved a maximum biofilm biomass reduction of 93.23 ± 1.20%, substantially exceeding the reductions observed with BA-FSE (80.42 ± 0.39%) and BA-CAE (40.85 ± 5.50%). These results highlight the superior biofilm-disrupting capacity of purified anthocyanin fractions and support their potential utility in antimicrobial formulations aimed at mitigating foodborne pathogens.

Lastly, study conducted by Omran et al. (2025) revealed anthocyanin extract from Plum (*Prunus domestica*) Peels exhibited significant antibacterial against Gram-negative bacteria. The antibacterial potential of the Prunus domestica peel extract was found to be concentration-dependent, with increasing extract concentrations correlating with larger inhibition zones. Specifically, inhibition zones measured 26.17 ± 0.42 mm for Serratia marcescens, 23.77 ± 0.42 mm for Bacillus cereus, 19.46 ± 0.50 mm for Pseudomonas aerogenes, 15.50 ± 0.42 mm for Staphylococcus aureus, 8.40 ± 0.42 mm for *Proteus mirabilis*, and 22.26 ± 0.42 mm for *Candida albicans*. These results suggest that anthocyanins from Plum peels possess broad-spectrum antimicrobial activity and may be particularly effective against certain Gram-negative and fungal pathogens.

## 9 Stability and bioavailability of anthocyanins

### 9.1 Stability of anthocyanins

Based on previous studies, anthocyanin compounds found in fruits of the Rosaceae family have been shown to possess a wide range of health-promoting properties. However, owing to their inherently unstable phenolic nature, anthocyanins are prone to degradation during food processing involving heat as well as throughout storage (Ayvaz et al., 2023). The structural instability of anthocyanins can significantly affect their biological activity. Their stability is influenced by various factors, particularly external conditions such as temperature, pH, radiation, the presence of enzymes and proteins, metal ions, and exposure to oxygen, moisture, light, and microorganisms. These factors may lead to anthocyanin degradation and a partial or complete decline in performance, including reduced color intensity and diminished fixation capacity within target matrices (Feitosa et al., 2023).

Temperature is one of the critical factors influencing the structural stability of anthocyanins. In general, exposure to high temperatures is known to cause significant degradation and a reduction in anthocyanin content. Thermal degradation of anthocyanins typically begins at around 60 °C. In the initial phase, anthocyanins undergo structural transformation into chalcones, followed by hydrolytic opening of the pyrylium ring. In the later stages, this process leads to the formation of insoluble polyphenolic compounds. Light is another critical factor that directly influences and accelerates anthocyanin degradation during storage. In addition to temperature and light exposure, storage duration also plays a significant role in determining anthocyanin stability (Ayvaz et al., 2023). Furthermore, the presence of oxygen during storage contributes to degradation processes, either through direct oxidative mechanisms or via the activity of oxidative enzymes such as polyphenol oxidase and peroxidase, which can damage the phenolic structure of anthocyanins and reduce their stability and biological efficacy. These factors are directly correlated with the decline in anthocyanin content and color intensity, reflecting a reduction in pigment quality during storage (Ayvaz et al., 2023).

In addition to environmental factors, anthocyanin degradation can also be attributed to the intrinsic characteristics of its molecular structure. An increased number of hydroxyl groups on the anthocyanin structure tends to reduce its stability, whereas the addition of methoxy groups can enhance it. Moreover, hydroxylation on the B-ring may trigger anthocyanin degradation into phenolic and aldehyde derivatives (Ayvaz et al., 2023). The theory regarding the influence of B-ring substituents on anthocyanin stability is supported by studies showing that pelargonidin-based anthocyanidins, with only one hydroxyl group on the B-ring, are the most stable. This is followed by malvidin, which contains two methoxy groups and one hydroxyl group. In contrast, the least stable anthocyanidins are delphinidin, which has three hydroxyl groups, and cyanidin, which has two hydroxyl groups on the B-ring (Tarone et al., 2020).

### 9.2 Bioavailability of anthocyanins

Anthocyanins have garnered considerable attention due to their substantial potential in the field of health. Unfortunately, they exhibit very low bioavailability. Research has shown that less than 2% of anthocyanins are effectively absorbed through the gastrointestinal tract (Rosales & Fabi, 2022). The low bioavailability of anthocyanins is also closely related to the polarity of these compounds. Based on their chemical structure and the attached functional groups, anthocyanins are highly polar molecules with extremely low solubility in lipids. The presence of various hydroxyl, sugar, and acyl groups significantly influences the molecular size, spatial conformation, and polarity of anthocyanins, ultimately determining the extent to which these compounds can be absorbed by the human body (Braga et al., 2018; Tarone et al., 2020). These characteristic limits their ability to penetrate cellular membranes, which are composed of phospholipid bilayers, thereby reducing their efficiency of cellular absorption (Xue et al., 2023). This is supported by research findings indicating that anthocyanins derived from pelargonidin, which contains only one hydroxyl group on the B-ring, are more readily absorbed compared to those with a greater number of substituents on the B-ring, such as peonidin, delphinidin, and cyanidin (Saini et al., 2024). As a result, the effectiveness of anthocyanins in the body is significantly hindered.

The absorption process of anthocyanins begins in the oral cavity. Human oral epithelial cells and terminal ducts of the salivary glands secrete hydrolases, phase II enzymes such as uridine diphosphate glucuronosyltransferase, and efflux transporters that support local intestinal circulation, resembling those found in the small intestine. These enzymes contribute to the degradation of anthocyanins into various metabolites. The enzymes related to oral endocrine enter the alimentary canal with anthocyanins, which makes anthocyanins further digested and absorbed (Xue et al., 2023). However, oral digestion of anthocyanins is limited due to their brief residence time in the oral cavity. Enzymes associated with oral endocrine activity enter the gastrointestinal tract along with anthocyanins, facilitating further digestion and absorption. The main metabolites of anthocyanins identified in the oral cavity were protocatechuic acid, phloroglucinaldehyde and anthocyanin glucuronide conjugates (Kamonpatana et al., 2012).

Anthocyanins that reach the stomach exhibit structural stability due to the acidic environment, with pH values typically ranging from 0.9 to 1.5. Under these conditions, anthocyanins predominantly exist in the flavylium cation (AH^+^) form. Approximately 1–10% of anthocyanins are absorbed by gastric epithelial cells in their intact molecular structure. Additionally, 10–20% of anthocyanins are actively transported into the bloodstream through gastric epithelial cells as primary metabolites (Han et al., 2019). Although the stomach is not generally considered a major site for nutrient absorption, several studies have demonstrated that anthocyanins can permeate the gastric wall and be absorbed without significant structural modification (Peixoto et al., 2016). According to studies conducted by (Oliveira, Perez-Gregório, et al., 2019; Oliveira, Roma-Rodrigues, et al., 2019), glucose transporter 1 (GLUT1) and glucose transporter 3 (GLUT3) have been identified as effective glucose transporters involved in the gastric absorption of anthocyanins.

Anthocyanin absorption primarily occurs in the intestinal tract through both passive and active transport mechanisms mediated by intestinal epithelial cells (Han et al., 2019). Fundamentally, this process involves the interaction between anthocyanins and epithelial cells via these dual transport pathways. Glycosylated anthocyanins can be transported and absorbed with the assistance of glucose transporters such as Sodium-Glucose Linked Transporter 1 (SGLT1) and glucose transporter (GLUT2). In addition, enzymes such as β-glucosidase and lactase-phlorizin hydrolase facilitate the cleavage of glycosidic bonds or the breakdown of the C-ring, resulting in the formation of low-molecular-weight phenolic acids that are more readily (Barik et al., 2020).

The remaining anthocyanin compounds and their intermediate metabolites subsequently pass from the small intestine to the colon, where they may undergo further absorption or microbial metabolism. The complex physiological conditions and diverse microbial flora in the colon can disrupt the ring structure of anthocyanins and degrade them into simpler phenolic acids, such as vanillic acid and hippuric acid, which are more readily absorbed by epithelial cells and subsequently enter the bloodstream. Upon entering systemic circulation, anthocyanins are distributed to target tissues to exert their biological functions. Unabsorbed anthocyanin residues are either transported to the liver, serving as a central site for further biotransformation, or eliminated via fecal, renal, or biliary excretion (Henriques et al., 2020).

## 10 Preservation methods for anthocyanin extracts in food applications

### 10.1 Encapsulation

Encapsulation has emerged as a prominent strategy for enhancing the stability and functionality of anthocyanins against environmental and physicochemical stressors that compromise their structural integrity and reduce their biological efficacy. Encapsulation is a technique or process by which core materials or bioactive compounds are entrapped within an immiscible solid or liquid matrix, commonly referred to as a carrier or wall material. This matrix acts as an effective physical barrier against environmental stressors that may compromise the structural integrity and biological functionality of sensitive compounds (Ghosh et al., 2021). Encapsulation methods can be classified based on the size of the resulting capsule particles. The particles, which may be in the form of dispersions or powders, are categorized as macrocapsules (>5000 μm), microcapsules (1.0–5000 μm), or nanocapsules (<1.0 μm) (Tarone et al., 2020). These strategies not only reduce the rate of degradation but also techniques enhance bioavailability by protecting anthocyanin molecules from extreme conditions within the gastrointestinal tract, thereby allowing these compounds to reach the small intestine intact, where absorption into the bloodstream occurs (Rocha et al., 2023; Saini et al., 2024).

In the food industry, several critical factors must be considered when designing encapsulation systems. These systems should be formulated using cost-effective ingredients and approved food-grade materials to ensure economic feasibility. Selecting an appropriate encapsulating agent requires careful evaluation of several key characteristics, including affinity with the core material, film-forming ability, biodegradability, resistance to gastrointestinal conditions, viscosity, solid content, hygroscopic properties, cost, and compatibility with the chosen encapsulation technique (Tarone et al., 2020). These factors are pivotal to the success of the encapsulation method and must be thoroughly assessed during the design phase (Ghosh et al., 2021).

Encapsulation techniques utilizing biopolymers, such as polysaccharides, proteins, and lipids, have demonstrated considerable potential for effectively enclosing anthocyanin-rich extracts. These biopolymers possess essential biological properties, including low toxicity, biodegradability, and compatibility with living tissues. Their natural recognition by human cells facilitates the targeted and controlled release of bioactive compounds in response to specific physiological signals (Rosales & Fabi, 2022). Polysaccharides are rich in hydrogen-bond donors and acceptors, such as ether linkages and hydroxyl groups, which make them highly suitable for binding polar molecules. These functional groups promote the formation of hydrogen bonds with anthocyanins, resulting in the creation of stable polysaccharide–anthocyanin complexes (Fu et al., 2024). Protein-based encapsulation has attracted growing interest in fields such as food science and medicine due to its versatility and effectiveness in delivering bioactive compounds. This approach offers a promising strategy for protecting and transporting active substances across various applications, including drug delivery, food technology, and healthcare systems (Rahul et al., 2024). Lipid materials also gained significant attention in the food industry due to their ability to enhance the bioavailability of bioactive compounds. Lipids are especially promising for encapsulating anthocyanins, a class of natural pigments with antioxidant properties, due to their low toxicity, biocompatibility, and scalability (Rosales & Fabi, 2022).

Numerous studies have explored the encapsulation of anthocyanins from natural sources, for instance, a study by Seke et al. (2023) demonstrated that microencapsulation of strawberry juice using pea protein isolate, psyllium mucilage, and okra mucilage significantly improved both thermal stability and bioaccessibility of pelargonidin-3-glucoside, the predominant anthocyanin, increased from 46.33 ± 0.55% to 56.33 ± 0.73% after simulated intestinal digestion. The resulting microcapsules remained relatively stable under physiological conditions, although substantial mass loss was observed at approximately 280 °C. These findings underscore the potential of such microcapsules as natural red colorants in food products. Complementary research by Rosales et al. (2023) focused on nanoencapsulation of blackberry anthocyanins using a pectin-based matrix associated with lysozyme via molecular self-assembly. The resulting nanoparticles, primarily containing cyanidin-3-O-glucoside, exhibited spherical morphology, high encapsulation efficiency (79%), and excellent physicochemical properties and zeta potential of 25.8 mV. These nanocapsules demonstrated enhanced thermal and gastrointestinal stability, with no cytotoxic effects observed at a 5% concentration, confirming their biocompatibility for food applications.

Further advancements were reported by Barba-Ostria, Carrera-Pacheco, et al. (2024) and Barba-Ostria, Gonzalez-Pastor, et al. (2024), who microencapsulated anthocyanin extracts from *Rubus glaucus* using maltodextrin through a spray-drying process. The results demonstrated enhanced bioactivity of the microencapsulated anthocyanins, suggesting their potential application in the development of functional foods and pharmaceuticals. This study demonstrates that microencapsulation effectively preserves the functional properties of anthocyanins and enhances their health-promoting benefits. The microencapsulated extracts of *Rubus glaucu*s exhibited diverse biological activities, including antioxidant, antibacterial, anticancer, and anti-inflammatory effects.

### 10.2 Copigmentation

Copigmentation reactions represent one of the most effective and practical approaches for enhancing anthocyanin stability through molecular interactions with non-colored phenolic compounds. These reactions improve anthocyanin stability by forming noncovalent complexes, which help preserve pigment integrity under various environmental and processing conditions (Ma et al., 2022. Copigmentation not only improves the stability of anthocyanins, but also enhances their antioxidant capacity and color intensity (Trouillas et al., 2016). The increase in color intensity is particularly important for natural colorants, as it enables a more visually appealing appearance with a reduced concentration of added pigments, thereby lowering production costs when applied at an industrial scale (Moshfegh et al., 2025).

The effectiveness of copigmentation is influenced by several factors, including the type and concentration of both the copigment and anthocyanin, their respective chemical structures, as well as the pH and temperature of the medium (Sari, 2016). Structurally, copigments are classified into phenolic and non-phenolic groups. The phenolic group comprises hydrolyzable tannins, flavonoids, and phenolic acids, while the non-phenolic group includes alkaloids, amino acids, organic acids, nucleotides, and polysaccharides (Trouillas et al., 2016). Flavonoids and phenolic acids are frequently utilized as copigmentation agents for anthocyanins extracted from various plant sources due to their extended π-conjugated systems and the presence of hydrogen bond donors and acceptor groups. Copigments possessing these structural features tend to exhibit higher efficiency, as such properties are known to facilitate noncovalent interactions, including Van der Waals forces, π−π stacking, hydrophobic interactions, and ionic bonding (Ma et al., 2022).

According to study conducted by Wu et al. (2025), the copigmentation between organic acids and cyanidin-3-glucoside (C3G) in blackberry wine was investigated to provide innovative insights into reducing browning during fruit wine aging and to offer theoretical guidance for mitigating environmental pollution caused by the spoilage of uneaten fruits. The addition of organic acids did not significantly alter the molecular weight of compounds in the solution. However, during copigmentation, organic acids modified the solution polarity, which induced rotation of the B-ring plane of C3G, restored shielding color intensity, and enhanced the color rendering strength of the C3G solution. Hydrophobic interactions and hydrogen bonding were identified as the main intermolecular forces influencing copigmentation. This study enriches the understanding of organic acid copigmentation on anthocyanins and provides a valuable reference for producing high-quality fruit wines while reducing environmental pollution from perishable fruits.

Similarly, Sun et al. (2025) employed citric and tannic acid as co-pigments to reduce color degradation in strawberry juice during thermal processing. Both compounds showed notable color stabilization, with optimal effects observed at a concentration of 4 mg/mL. Thermal treatment at 60–80 °C enhanced anthocyanin stability by lowering degradation rates, with tannic acid demonstrating greater co-pigmentation efficacy than citric acid. This suggests that tannic acid provides stronger stabilization of anthocyanins and is more suitable for combination with thermal sterilization to minimize color loss in strawberry juice during industrial production. Furthermore, Salati et al. (2025) examined the combined effects of co-pigmentation on the physicochemical properties and storage stability of anthocyanin powders, as well as their performance in a model beverage system. Anthocyanin powders co-pigmented and encapsulated with a maltodextrin–arabic gum (MD–AG) matrix exhibited a significantly more intense red coloration. After 28 days of storage under three different conditions, the co-pigmented samples retained markedly higher anthocyanin levels compared to the non-co-pigmented controls. In model beverages, color stability was preserved for up to 49 days in both encapsulated powders and control samples (anthocyanin extracts with or without TA). Overall, co-pigmentation with TA combined with MD–AG encapsulation provides an effective strategy for enhancing the stability and color retention of sour cherry anthocyanins.

## 11 Conclusion

Rosaceae fruits have been shown to possess diverse anthocyanin structures with a wide range of associated bioactivities. A comprehensive review identified 37 distinct anthocyanin compounds from Rosaceae fruits, comprising 17 cyanidin derivatives, 7 delphinidin, 6 pelargonidin, 3 peonidin, and 2 each from the malvidin and petunidin groups. Anthocyanins derived from cyanidin, delphinidin, and pelargonidin were the most frequently detected, suggesting the presence of active biosynthetic pathways involving hydroxylation at the 3′ and 5′ positions of the flavonoid B-ring, catalyzed by F3′H and F3′5′H. In addition to variations in anthocyanidin backbone structures, anthocyanin diversity is further influenced by the variety of sugar moieties attached to the core molecule. The most commonly identified sugar groups in anthocyanins from Rosaceae fruits include both monosaccharides and disaccharides. Among the monosaccharides, glucoside, galactoside, and arabinoside were predominant, while rutinoside, sambubioside, and sophoroside represented the major disaccharide forms. Furthermore, several anthocyanins underwent acylation, resulting in acylated derivatives such as malonyl-glucoside, acetyl-glucoside, and succinyl-glucoside. These structural modifications indicate that enzymes UFGT and AAT play key roles in anthocyanin biosynthesis through glycosylation and acylation mechanisms. In vitro and in vivo studies have demonstrated that consumption of Rosaceae fruits confers multiple biological activities, including antioxidant, anti-inflammatory, anti-obesity, antidiabetic, anticancer, and antibacterial effects. Additionally, anthocyanin-rich extracts from Rosaceae fruits have potential applications as natural food colorants, offering a safer alternative to synthetic dyes known to pose health risks. The functionality of anthocyanin extracts can be further enhanced through encapsulation or copigmentation techniques, which not only improve their efficacy but also protect anthocyanin structures from degradation caused by environmental and chemical stressors. Despite these advantages, the large-scale application of anthocyanin-rich extracts from Rosaceae fruits as natural colorants in the food industry still faces several challenges, including the establishment of safety evaluation standards, quality control measures, regulatory approval of health claims, and industrial-scale production. To address these issues, future research should prioritize the standardization of extraction and characterization protocols, the strengthening of toxicological and bioavailability data, and the execution of well-designed clinical studies to support regulatory approval.

## CRediT authorship contribution statement

**Muhammad Habibul Ikhsan:** Writing – original draft, Methodology, Investigation, Data curation. **Selvi Apriliana Putri:** Writing – original draft, Visualization. **Herlandita Rona Anggraeni:** Visualization. **Rika Septiyanti:** Writing – original draft. **Ari Hardianto:** Writing – review & editing, Supervision. **Jalifah Latip:** Supervision. **Tati Herlina:** Writing – review & editing, Supervision, Conceptualization.

## Declaration of competing interest

The authors declare that they have no known competing financial interests or personal relationships that could have appeared to influence the work reported in this paper.

## Data Availability

Data will be made available on request.
