# Supplementary material for: Anthocyanins from Rosaceae fruits: diversity, bioactivity, and potential as natural colorants
Source: Food Chem X. 2026 Jan 29;34:103606. doi: 10.1016/j.fochx.2026.103606 (PMC12887364; doi:10.1016/j.fochx.2026.103606)
Supplement: Supplementary material [file mmc1.docx]

**Fig S1.** Anthocyanins Structure from Rosacea Fruits
